# Melatonin Mitigates Central Sensitization and Nociplastic Pain in Spinal Cord and Dorsal Root Ganglia of FM Rat Model: Modulation of SIRT1/PGC-1α/MAPK/NF-κB Signaling

**DOI:** 10.1007/s11481-025-10274-7

**Published:** 2026-02-13

**Authors:** Jehad Osama, Amira A. El-Gazar, Ghada M. Ragab, Nesrine S. EL-Sayed, Ahmed S. Kamel

**Affiliations:** 1 Postgraduate program in Pharmacology & Toxicology, Faculty of Pharmacy, Cairo University, Giza, Egypt; 2 Department of Pharmacology & Toxicology, Faculty of Pharmacy, Cairo University, Giza, Egypt; 3 Department of Pharmacology & Toxicology, Misr University for Science and Technology, Giza, Egypt; 4 Department of Pharmacology & Toxicology, October 6 University, Giza, Egypt

**Keywords:** Fibromyalgia, Melatonin, Glutamatergic dysregulation, Dorsal root ganglia, Spinal cord, Mitochondria

## Abstract

Fibromyalgia (FM) is a central sensitization syndrome characterized by neuroinflammation and synaptic hyperexcitability, which amplifies pain signals in the spinal cord (SC) and dorsal root ganglia (DRG). Melatonin (MEL) has demonstrated analgesic and antinociceptive properties in experimental models, supporting its clinical application in various pathological conditions. This study aimed to elucidate the impact of Melatonin on neuroinflammation and glutamatergic dysregulation in the spinal cord and DRG using a Reserpine-induced fibromyalgia model (RIFM). RIFM was induced in female rats by administering Reserpine (1 mg/kg/day, s.c.) for three consecutive days. Melatonin (10 mg/kg, orally) was administered for three days following the peak of pain on day 7. Melatonin significantly ameliorated stimulus-evoked pain in thermal and mechanical tests, with a resolution of spontaneous pain noted by the rat grimace scale. Moreover, motor activity in the open field task and depressive behavior in the tail suspension test (TST) were alleviated following Melatonin administration. Fibromyalgia pain was associated with enhanced glutamatergic transmission, as shown by immunohistochemical assessment of synaptophysin and PSD95 in the DRG along with VGLUT, PSD95, NMDA, NMDA receptor 2B, and AMPA in the spinal cord. Reserpine-induced disturbances in mitochondrial biogenesis markers, SIRT1 and PGC-1α, were followed by an upsurge of TNF-α, NFkB, and P38-MAPK. This neuroinflammatory milieu was marked by elevated ionized calcium-binding adaptor molecule-1 (Iba-1) in activated microglia within the spinal cord. These pathological findings were notably mitigated by Melatonin, as reflected by increased expressions of spinal MT1 and MT2 receptors. In conclusion, Melatonin exhibited antinociceptive and anti-inflammatory effects by modulating glutamate neurotransmission, mitochondrial dysfunction, and microglial activation, thereby alleviating nociplastic pain in the experimental RIFM.

**Graphical Abstract:**

The potential role of Melatonin in modulating Reserpine-induced fibromyalgia in rats through MT1R and MT2R receptors

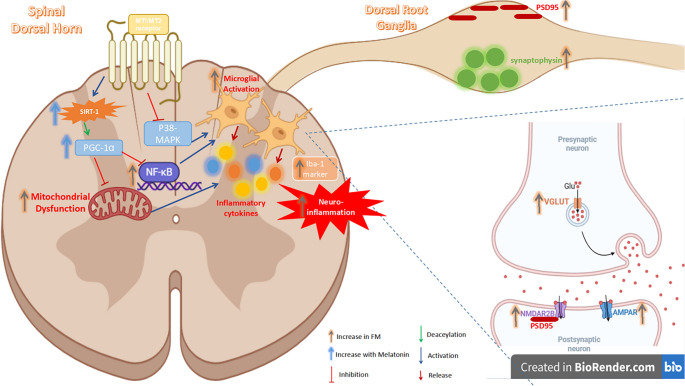

**Supplementary Information:**

The online version contains supplementary material available at 10.1007/s11481-025-10274-7.

## Introduction

Fibromyalgia (FM) syndrome, the most common central sensitivity syndrome (CSS), affects approximately 2–5% of the population (Boomershine 2015; Sarzi-Puttini et al. 2020). It is a persistent disease that is distinguished by severe pain in the muscles and joints, with mechanical and thermal allodynia being common among FM patients. FM typically occurs in middle-aged women at a ratio of 3:1, although it can affect both sexes at any age (Brum et al. 2020). There are multiple contributing factors, such as central sensitization and neuroinflammation mediated by cytokines and glial activation. Additional factors include autonomic dysregulation, small fiber neuropathy, as well genetic and epigenetic predispositions such as polymorphisms in the catechol-O-methyltransferase well as alterations in pain processing pathways (Siracusa et al. 2021; Gyorfi et al. 2022).

In recent years, the International Association for the Study of Pain has acknowledged “nociplastic pain” as a tertiary mode of pain genesis. When there is no actual or anticipated tissue injury, nociplastic pain is defined as pain that results from altered nociception (Fitzcharles et al. 2021). Excitatory neurotransmitters like glutamate facilitate synaptic transmission within the dorsal horn and dorsal root ganglia (DRG), amplifying pain transmission through ascending pathways conveying nociceptive signals from the periphery to the brain (Latremoliere and Woolf 2009; Yam et al. 2018). Independent reviews confirm that mitochondrial dysfunction, activated glutamate receptors, and microglial activation in the spinal dorsal horn synergistically enhance ascending nociceptive signaling (Kung et al. 2013; Deng et al. 2019; Cao et al. 2024; Espinoza and Papadopoulos 2025). Additionally, the proximity of the DRG to the subarachnoid space may link central sensitization to the elevated levels of the pain-promoting neurotransmitter glutamate in the cerebrospinal fluid of FM patients, as observed through neuroimaging of the brain (Martínez-Lavín 2022).

Mitochondria play a vital role at the cellular level in fibromyalgia (FM) patients (Meeus et al. 2013; Brum et al. 2020; Macchi et al. 2024). In this context, mitochondrial dysfunction refers to impaired biogenesis and activity, often characterized by reduced expression of key regulatory proteins such as phosphorylated peroxisome proliferator-activated receptor gamma coactivator 1-alpha (PGC-1α) and Sirtuin 1 (SIRT1), both of which are essential for maintaining mitochondrial health. Evidence from clinical and preclinical studies indicates that mitochondrial dysfunction contributes to increased pain sensitivity (Brum et al. 2020; Jung et al. 2021). Moreover, mitochondrial dysregulation frequently triggers an inflammatory response through activation of pathways such as NF-κB and P38 MAPK, thereby exacerbating the neuroinflammatory environment (Qin et al. 2018; Castro et al. 2022). These molecular alterations foster neuroinflammation and facilitate pain sensitization in FM. The resulting disturbed environment promotes microglial activation, which is associated with glutamate release in the spinal dorsal horn, further amplifying central sensitization (Kawasaki et al. 2008; Domercq et al. 2013; Ji et al. 2018; Chen et al. 2018; Gu et al. 2022; Atta et al. 2023a; Kohno and Tsuda 2025). Microglial activation, a hallmark of neuroinflammation, can be identified by increased expression of ionized calcium-binding adapter molecule 1 (Iba-1), a calcium-binding protein specifically expressed in microglia with surge of proinflammatory cytokines (Gui et al. 2016; Qi et al. 2016; Vanderwall and Milligan 2019; Zhang et al. 2025). Thus, regulating mitochondrial function may suppress neuroinflammation by modulating microglial activation and glutamatergic dysregulation. In addition to DRG, the spinal dorsal horn showed glutamatergic changes in chronic pain through enhanced excitatory neurotransmission, viz., glutamatergic signaling. Upregulation of markers such as vesicular glutamate transporter (VGLUT), postsynaptic density protein 95 (PSD95), and ionotropic glutamate receptors (NMDA and AMPA) contributes to synaptic potentiation and central sensitization, critical for persistent pain (Liu and Salter 2010; Niciu et al. 2012; Bardoni 2013; Turan Yücel et al. 2023; Jang and Garraway 2024). Therefore, there is an urgent need for therapeutic interventions targeting mitochondrial dysfunction and synaptic hyperexcitability in FM patients.

Melatonin is a neurohormone that shows antinociceptive effects across various pain models. Melatonin exerts its actions primarily through its receptor subtypes, MT1 and MT2, which are expressed in the spinal cord (Gao et al. 2020; Xiong et al. 2024). These receptors have been extensively investigated in models of neuropathic pain, migraine, and irritable bowel syndrome (Srinivasan et al. 2012). Melatonin attenuated mitochondrial dysfunction and neuroinflammation via its receptors and downstream signaling pathways, including SIRT1 activation (Zeng et al. 2023). Moreover, SIRT1 mediated Melatonin’s inhibitory effects on microglial activation, further reducing neuroinflammation (Merlo et al. 2020). In models of nerve injury, Melatonin suppressed microglial activation via inhibiting the p38 MAPK signaling pathway that modulated behavioral hypersensitivity (Chiang et al. 2013). However, a gap remains in exploring Melatonin’s impact on mitochondrial dysfunction and neuroinflammation-induced central sensitization in FM.

Therefore, the present study aimed to examine melatonin’s potential to alleviate central sensitization and nociplastic pain in a reserpine-induced FM model by restoring mitochondrial function and attenuating the consequent neuroinflammatory and glutamatergic disturbances in the spinal cord and DRG. As well, investigating changes in MT1 and MT2 receptor expressions that provide insights into the mechanisms underlying Melatonin’s potential therapeutic effects in FM. To achieve this aim, FM was induced using reserpine (RES). The RES-induced fibromyalgia model (RIFM) is a well-established and extensively used animal model that replicates the etiological and clinical features of FM, encompassing (i) the cardinal symptoms experienced by FM patients, such as musculoskeletal pain, spontaneous nociception, and pain hypersensitivity; (ii) primary comorbidities, including fatigue, depression, sleep disturbances, and anxiety; and (iii) characteristic pathological mechanisms, such as peripheral and central sensitization, neuroinflammation, and alterations in the levels of excitatory and inhibitory neurotransmitters (Yao et al. 2020; Brum et al. 2022).

## Materials and methods

### Animals

Female Wistar rodents (160–200 g) were obtained from the animal facility of the Egyptian Drug Authority in Giza, Egypt. The study was designed to focus on female rats intentionally, as fibromyalgia (FM) affects women at a significantly higher rate, with approximately 80–96% of FM patients being female (Wolfe et al. 2010; Carson et al. 2010; Cabo-Meseguer et al. 2017; Ruschak et al. 2023). Before the experiment, the animals were subjected to laboratory conditions at the Faculty of Pharmacy, Cairo University’s animal facility, for a period of seven days. The rodents were confined in conventional polycarbonate enclosures (40 × 25 × 15 cm) in groups of five during the acclimatization and experimental phases. The environmental conditions were meticulously monitored, with a stable room temperature of 25 ± 2 °C, a 12-hour light/dark cycle (lights on from 7:00 AM to 7:00 PM), and a relative humidity of 60 ± 10%. In an effort to observe the rodents without substantially disrupting their nocturnal period, the lighting was meticulously controlled with faint red lights. In order to guarantee sanitary conditions, bedding was replaced daily, and cages were cleansed. The animals were provided with unlimited access to potable water and standard food pellets (National Research Centre diet, Giza, Egypt) during the course of the investigation. Furthermore, daily monitoring of room humidity and ambient temperature was implemented to mitigate environmental fluctuations that could potentially affect the experimental outcomes.

## Results

### MEL Alleviated Spontaneous- and Evoked-Pain Perception in the FM-Like Model

**Fig. 1:**
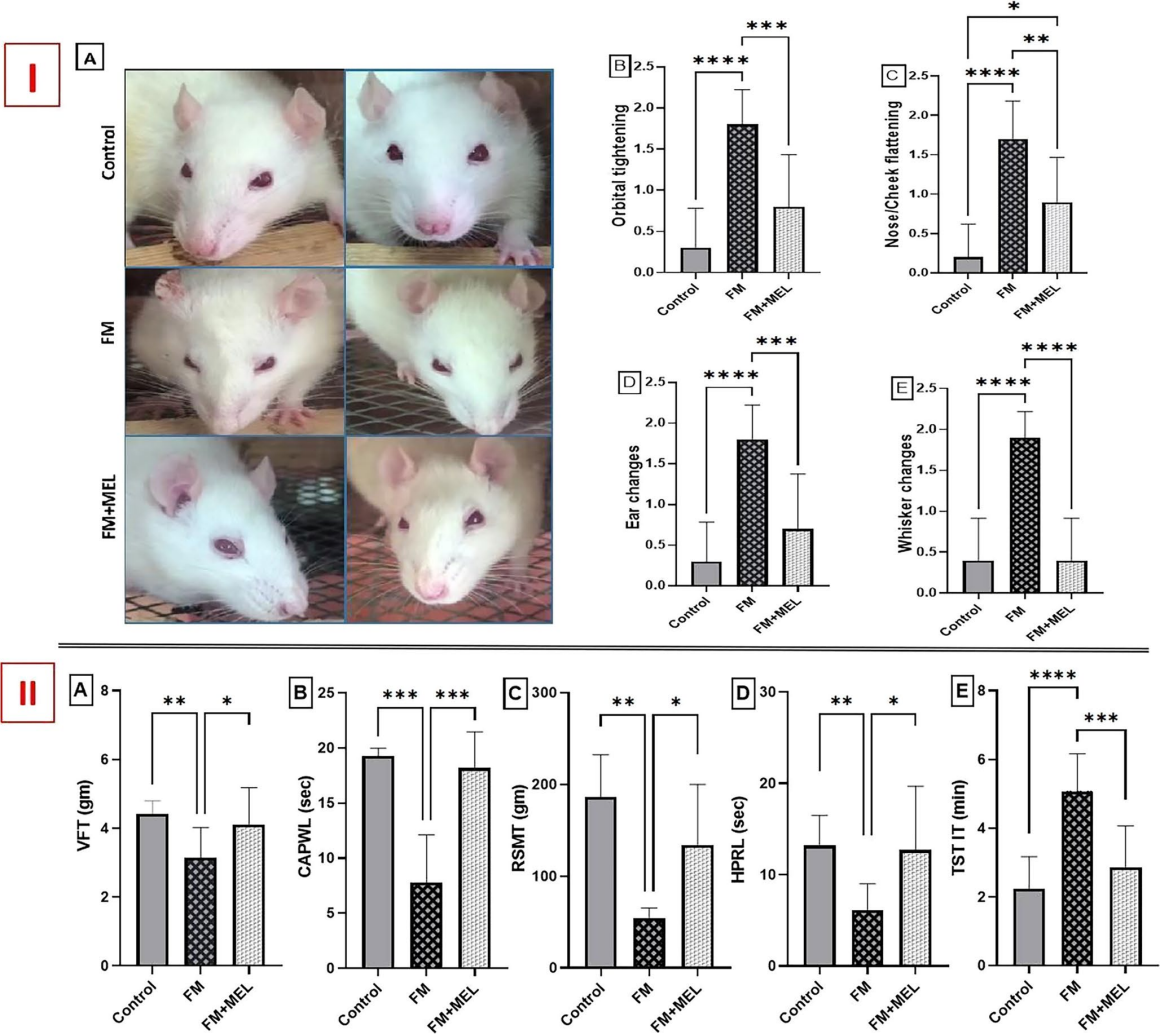
**(I)** Impact of Melatonin (10 mg/kg, oral, 3 days) on (**A**) facial expression-captured photos, (**B**) orbital tightening, (**C**) nose/cheek flattening, (**D**) ear changes, and (**E**) whisker changes tracking spontaneous pain in the FM-like model using the rat grimace scale. Using one-way ANOVA and Tukey’s post-hoc test (*p < 0.05, **p < 0.01, ***p < 0.001, and ****p < 0.0001), the mean ± SD of rats (*n* = 10) per group is indicated by each bar with a vertical line. **(II)** Impact of Melatonin (10 mg/kg, oral, 3 days) on FM rats using (A) VFT, (B) CAPWL, (C) RSMT, (D) HPRL, and (E) TST IT to measure nociceptive reflexes and hyperalgesic behaviors in response to the induced pain. Using one-way ANOVA and Tukey’s post-hoc test (*p < 0.05, **p < 0.01, ***p < 0.001, and ****p < 0.0001), the mean ± SD of rats (*n* = 10) per group is indicated by each bar with a vertical line. VFT: von Frey test; CAPWL: cold allodynia paw withdrawal latency; FM: fibromyalgia model; HPRL: hot plate reaction latency; MEL: Melatonin; RSMT: Randall-Sellito mechanical threshold; TST IT: tail suspension test immobility time

To assess whether Melatonin affects spontaneous pain —a key characteristic of FM —spontaneous facial expressions were recorded using the rat grimace scale, which was then quantified to measure spontaneous pain. Five major changes regarding the rat grimace scale were represented in Fig. 1(I): (A) facial expression-captured photos of different groups, (B) orbital tightening, (C) nose/cheek flattening, (D) ear changes, and (E) whisker changes. At *p* < 0.0001, the FM group revealed (A) eye closure or eye squeezing, (B) less bulging of the nose and cheek, (C) increased ear folding, curling, and outward angling, resulting in a pointed shape, and (D) whiskers moved forward away from the face, tending to appear as if standing on end, to reach six folds [F_(2,27)_ = 21.58], 8.5 folds [F_(2,27)_ = 23.05], six folds [F_(2,27)_ = 20.88], and 4.75 folds [F_(2,27)_ = 35.53], respectively, compared to the naïve rats.

In contrast, the treated group verified the substantial efficacy of Melatonin in reducing spontaneous pain, as reflected by (A) orbital tightening, (C) ear, and (D) whisker changes reaching the normal control scores. Meanwhile, FM rats treated with Melatonin only normalized (B) nose and cheek flattening by 47% compared to the FM group.

To evaluate the effect of Melatonin administration on mechanical and thermal pain sensitivity in RIFM, the study employed a series of behavioral assessments, such as the von Frey filament test, cold allodynia test, hot plate test, Randall-Sellito test, and tail suspension test, to comprehensively characterize pain responses associated with FM. These tests were chosen to measure different modalities of pain sensitivity, thereby providing a detailed understanding of Melatonin’s potential anti-nociceptive effects. Figure 1(II) demonstrates rats with FM induced by Reserpine presented an exacerbation in the urged pain response, as noted by the aberrations in the nociceptive reflexes through the decremented threshold of (A) the von Frey test (VFT) and (B) CAPWL, reaching 71% [F_(2,27)_ = 6.376, *p* = 0.0053, η^2^ = 0.321] and 40% [F_(2,27)_ = 20.12, *p* = 0.0003, η^2^ = 0.770], respectively, compared to the normal rats.

Furthermore, using the Randall-Sellito and hot plate tests, FM rats manifested a marked hindrance in the (C) Randall-Sellito mechanical threshold (RSMT) and (D) hot plate reaction latency (HPRL) by 71% [F_(2,27)_ = 10.06, *p* = 0.0021, η^2^ = 0.626] and 54% [F_(2,27)_ = 6.921, *p* = 0.0068, η^2^ = 0.339], respectively, relative to the healthy rats. Furthermore, a supraspinal pain response was revealed in the reserpinized rats, indicated by extended immobility time in (E) TST, reaching 2.27-fold [F _(2, 27)_ = 18.91, *p* = 0.0001, η^2^ = 0.583] compared to the vehicle group. Conversely, oral consumption of Melatonin for three days significantly boosted the withdrawal threshold to attain 1.3-fold and 2.3-fold in (A) VFT (*p* = 0.0412) and (B) CAPWL (*p* = 0.0006), respectively, unlike the FM group values. Additionally, Melatonin’s anti-allodynic effect was verified by increased pain endurance in (C) RSMT and (D) HPRL, achieving 2.48-fold and 2.08-fold relative to the Reserpine-induced FM rats. Ultimately, Melatonin treatment mitigated the pain and improved mobility to approach 56% relative to the untreated rats, at *p* = 0.0003. All these results are summarized in Fig. 1 (I) and (II), reflecting Melatonin’s antinociceptive effect.

### Melatonin Restored Motor Coordination in the FM-like Model

**Fig. 2:**
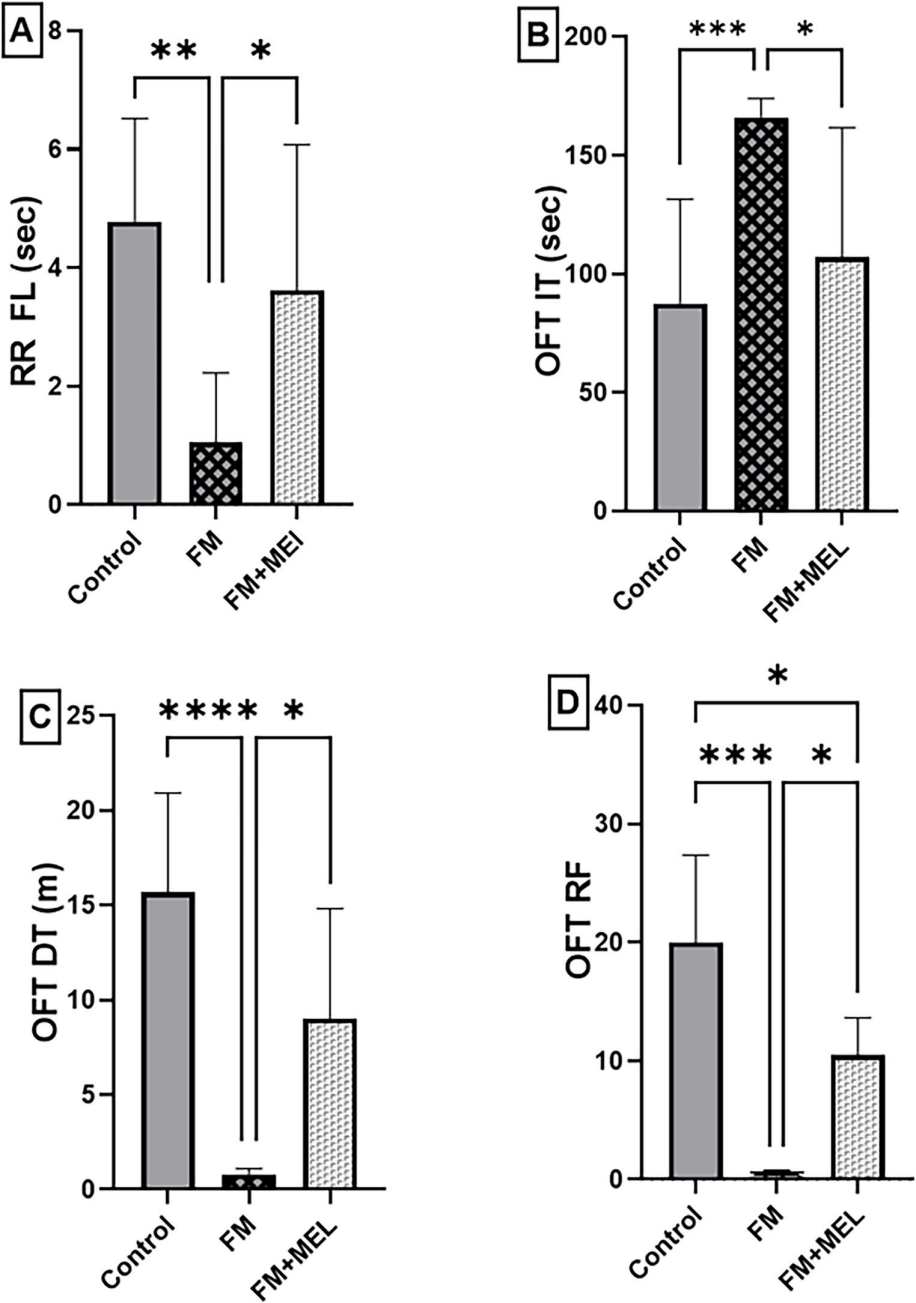
Impact of Melatonin (10 mg/kg, oral, 3 days) on locomotor activity and motor coordination by (**A**) RRFL using the Rota rod test and (**B**) OFT IT, (**C**) OFT DT, and (**D**) OFT RF using the open field test. Using one-way ANOVA and Tukey’s post-hoc test (*p < 0.05, **p < 0.01, ***p < 0.001, and ****p < 0.0001), the mean ± SD of rats (*n* = 10) per group is indicated by each bar with a vertical line. FM: fibromyalgia model, MEL: Melatonin, OFT DT: distance traveled in open field test, OFT IT: immobility time in open field test, OFT RF: rearing frequencies in open field test, RR FL: falling latency in the Rota rod test

To investigate whether Melatonin can modulate motor disturbances associated with the FM model, the Rota rod test and open-field test were employed. These assessments aimed to evaluate potential improvements in motor coordination and activity levels, thereby exploring additional therapeutic effects of Melatonin beyond pain modulation (Fig. 2). In the Rota rod test, Reserpine-induced FM drastically decreased (A) the rat’s retention time on the RR by 78% [F _(2, 27)_ = 8.312, *p* = 0.0016, η^2^ = 0.41] compared to the healthy rats. Regarding the open field test, FM rats revealed a substantial reduction in (B) open field test immobility time (OFT IT) by 90% [F_(2,27)_ = 11.64, *p* = 0.0002, η^2^ = 0.492] and (C) open field test distance traveled (OFT DT) by 95% [F_(2,27)_ = 23.03, *p* < 0.0001, η^2^ = 0.767], contrasted with healthy ones. In addition, the subcutaneous administration of RES effectively aggravated the neuropathic pain reported herein by reducing the (D) rearing frequencies in the open field test (OFT RF) in the FM group by 99% [F_(2,27)_ = 22.87, *p* = 0.0001, η^2^ = 0.821]. Contrarily, Melatonin prolonged (A) RIFM’s falling latency by 77% (*p* = 0.0279) and effectively countered the motor alterations manifested by reserpinized rats in the OFT, resulting in 0.65-fold for OFT IT (*p* = 0.0169), 11.46-fold for OFT DT (*p* = 0.0104), and a notable rise in vertical upright behavior by 51.5-fold (*p* = 0.0143).

### Melatonin Mitigated Histological Alterations in the DRG Within the FM-like Model

**Fig. 3:**
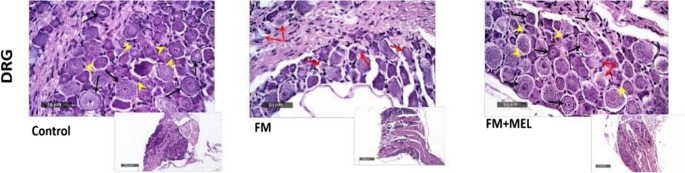
Impact of Melatonin (10 mg/kg, oral, 3 days) on histopathological changes in the FM rat model. Photomicrographs of H&E staining of the DRG are shown. Black arrows indicate intact, well-organized neurons with preserved subcellular details; the yellow arrowhead highlights sporadic degenerated neurons, well-organized satellite ganglionic cells, and Schwann cells exhibiting intact myelinated nerve fibers. The red arrow points to degenerated neurons with nuclear pyknosis, occasional chromatolysis, wider interneuronal spaces, and marked perineuronal ganglionic satellite cell loss. DRG: dorsal root ganglia, FM: fibromyalgia, MEL: Melatonin. Scale bar: 200 and 50 µM

Normal control samples exhibited intact neuronal types of various sizes with preserved subcellular details (black arrow), few sporadic degenerated neurons, well-organized satellite ganglionic cells (yellow arrowhead), and Schwann cells with intact myelinated nerve fibers. In contrast, FM samples showed focal areas of degenerated and shrunken neurons with nuclear pyknosis and occasional chromatolysis (red arrow), accompanied by wider interneuronal spaces and a significant loss of perineuronal ganglionic satellite cells. However, FM + Melatonin rats demonstrated neuroprotective efficacy, as evidenced by an increased number of intact neurons (black arrow) similar to those in normal control samples, moderate restoration of satellite ganglionic cell densities (yellow arrowhead), and a reduced extent of nerve damage (red arrows) (Fig. 3).

### Melatonin up-regulated the MT1R and the MT2R in the Spinal Cord of RIFM

**Fig. 4:**
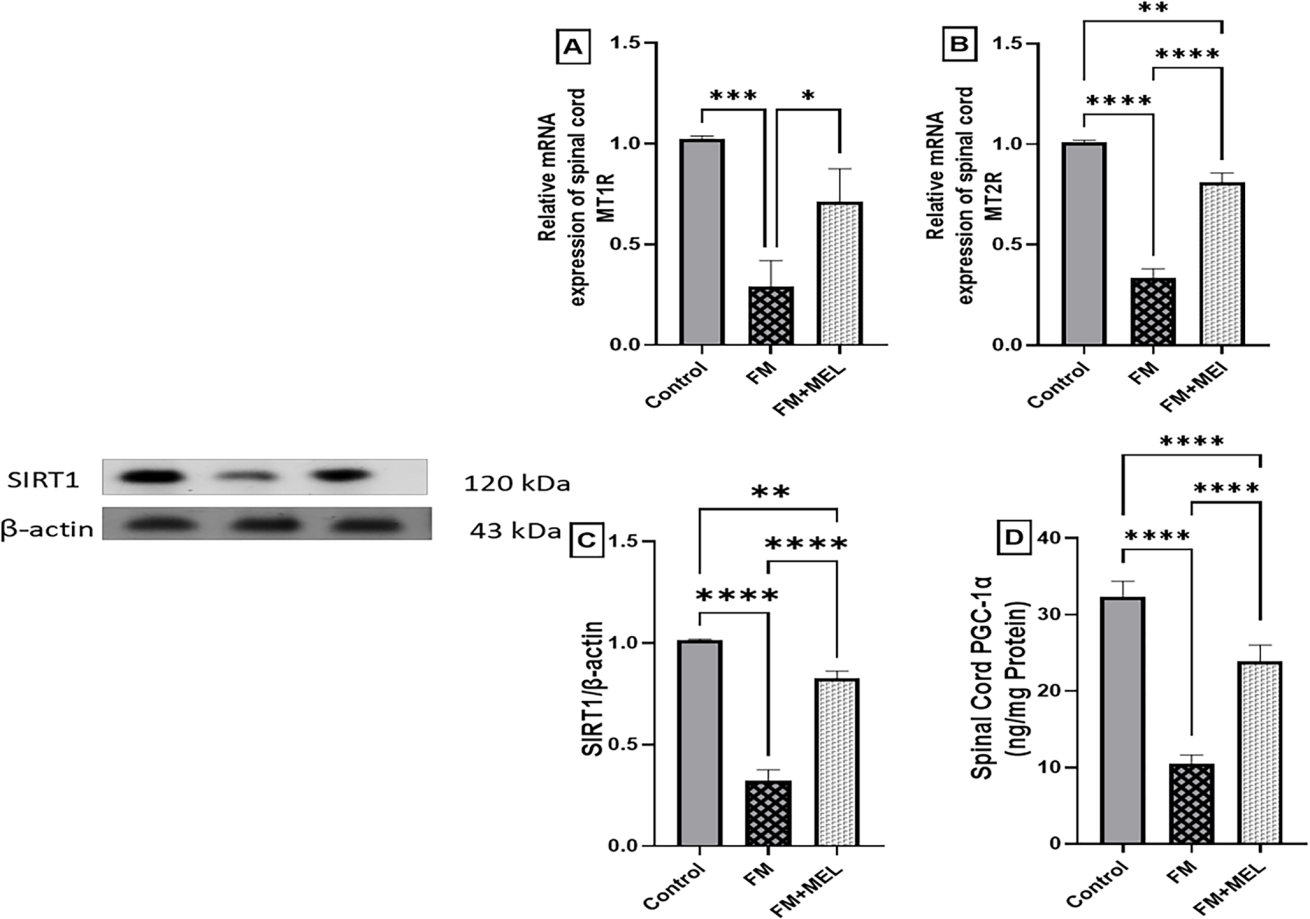
Impact of Melatonin (10 mg/kg, oral, 3 days) on mRNA expression of the spinal cord (**A**) MT1R and (**B**) MT2R in the FM-like model. Using one-way ANOVA and Tukey’s post-hoc test (*p < 0.05, **p < 0.01, ***p < 0.001, and ****p < 0.0001), the mean ± SD of rats (*n* = 3) per group is indicated by each bar with a vertical line. MEL: Melatonin, FM: fibromyalgia model, MT1R: Melatonin receptor 1, and MT2R: Melatonin receptor 2. In addition to the impact of Melatonin on the spinal cord, (**C**) SIRT1 protein expression and (**D**) PGC1α protein content indicate mitochondrial biogenesis in the FM-like model. The mean ± SD of rats (*n* = 3 for SIRT1 and *n* = 6 for PGC1α per group) is represented by each bar with a vertical line. Analyses were conducted using one-way ANOVA and Tukey’s post-hoc test (*p < 0.05, **p < 0.01, ***p < 0.001, and ****p < 0.0001). FM: fibromyalgia model, MEL: Melatonin, PGC1α: peroxisome proliferator-activated receptor gamma coactivator 1-alpha, SIRT1: silent information regulator sirtuin 1

To explore the role of Melatonin receptors in mediating its anti-nociceptive effects, gene expression levels of MT1R and MT2R in the rat FM spinal cord were measured using quantitative polymerase chain reaction. This analysis aimed to determine whether alterations in receptor expression contribute to Melatonin’s antinociceptive properties. Reserpine administration drastically reduced MT1R (F_(2, 6)_ = 27.99, *p* = 0.0009, η^2^ = 0.903) and MT2R (F_(2, 6)_ = 245.3, *p* < 0.0001, η^2^ = 0.988) in the spinal cord by 72% and 67%, respectively, compared to normal mRNA expression in healthy rats. Melatonin significantly increased MT1R and MT2R mRNA expression in treated animals to 70% and 80% of the FM group, as shown in Fig. 4 A and B.

### Melatonin Ameliorated Mitochondrial Dysfunction in the Spinal Cord of RIFM

To investigate whether Melatonin influences mitochondrial biogenesis in FM, the expression levels of SIRT-1 and PGC-1α were measured. These markers were selected to evaluate potential mitochondrial effects of Melatonin in the context of FM. Dysfunction in mitochondrial biogenesis was confirmed in FM rats through a reduction in the spinal cord (C) Sirt1 protein expression by 68% (F _(2, 6)_ = 265.3, *p* < 0.0001, η^2^ = 0.989) and (D) PCG1α protein content by 67% (F _(2, 15)_ = 218.5, *p* < 0.0001, η^2^ = 0.967), as shown in Fig. 4. Melatonin treatment resulted in an increase in (C) Sirt1 protein expression (2.5-fold) and (D) PCG1α protein content (2.28-fold) compared to the FM group (Fig. 4 C and D).

### Melatonin Restrained Neuroinflammatory Mediators, MAPK and NF-κB, in the Spinal Cord of RIFM

**Fig. 5:**
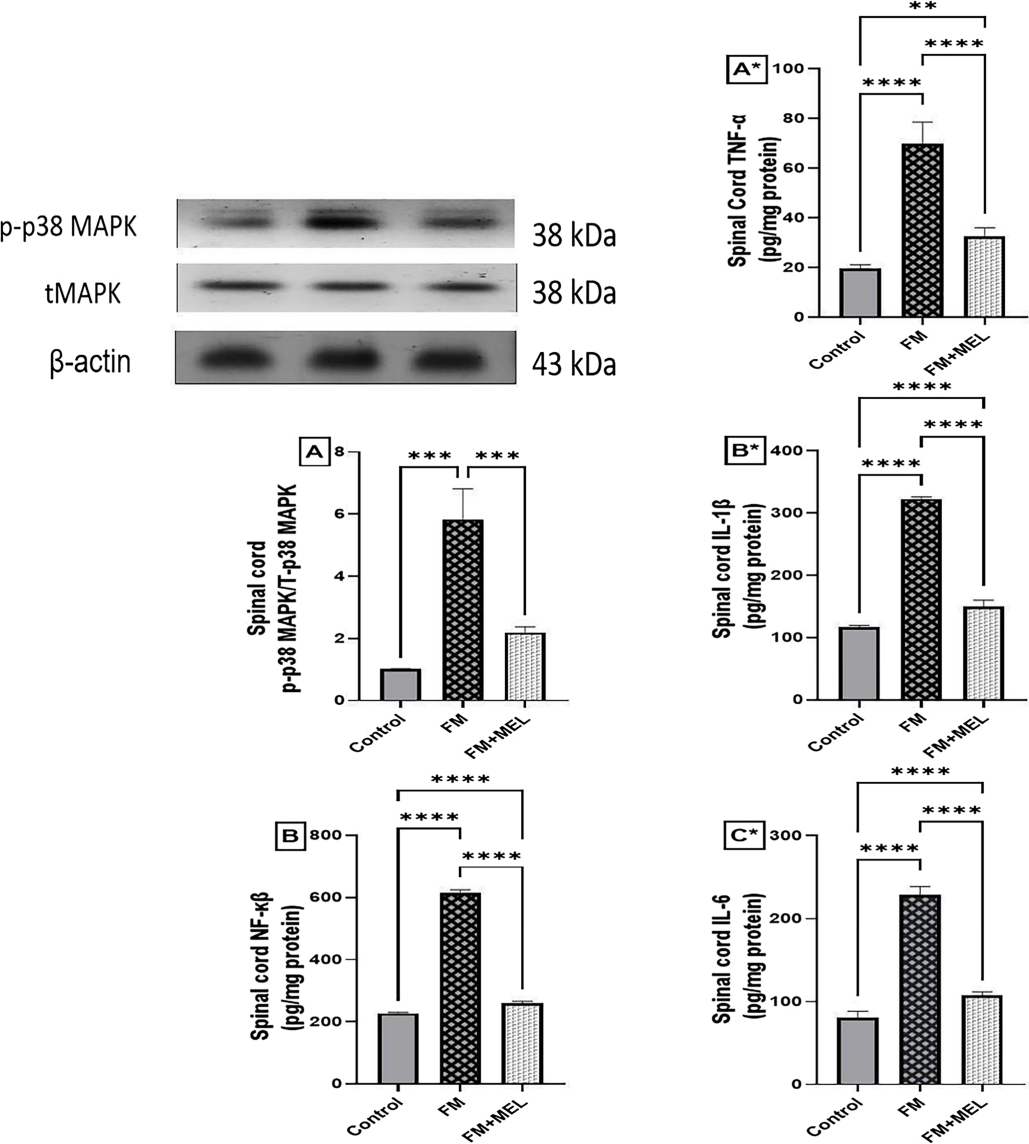
Impact of Melatonin (10 mg/kg, oral, 3 days) on spinal cord pro-inflammatory modulators (**A**) p-p38 MAPK/total p38-MAPK and (**B**) NF-κB in the FM-like model. Using one-way ANOVA and Tukey’s post-hoc test (*p < 0.05, **p < 0.01, ***p < 0.001, and ****p < 0.0001), the mean ± SD of rats (*n* = 3 for MAPK and *n* = 6 per group for NF-κB) is represented by each bar with a vertical line. FM: fibromyalgia model, MAPK: mitogen-activated protein kinases, MEL: Melatonin, NF-κB: nuclear factor kappa B. Impact of Melatonin (10 mg/kg, oral, 3 days) on spinal cord pro-inflammatory cytokines (A*) TNF-α, (B*) IL-1β, and (C*) IL-6 in the FM-like model. Using one-way ANOVA and Tukey’s post-hoc test (*p < 0.05, **p < 0.01, ***p < 0.001, and ****p < 0.0001), the mean ± SD of rodents (*n* = 6) per group is indicated by each bar with a vertical line. FM: fibromyalgia model, IL-1β: interleukin-1 beta, IL-6: interleukin-6, MEL: Melatonin, TNF-α: tumor necrosis factor

To evaluate the anti-neuroinflammatory effects of Melatonin, the levels of key neuroinflammatory mediators, p-MAPK and NF-κB, were measured. These markers help determine the extent to which Melatonin modulates neuroinflammation in FM. As depicted in Fig. 5 A, phosphorylation of MAPK protein expression significantly increased in FM rats, reaching 5.78-fold [F _(2, 6)_ = 56.53, *p* = 0.0001, η^2^ = 0.949] compared to the healthy group. Furthermore, Fig. 5B shows that FM rats exhibited a 2.7-fold increase in NF-κB levels [F _(2, 15)_ = 6357, *p* < 0.0001, η^2^ = 0.998] compared to naïve rats. Conversely, Melatonin oral treatment inhibited p38MAPK phosphorylation, reducing it by 38% compared to reserpinized rats. Additionally, Melatonin-treated rats showed a reversal effect on NF-κB levels, decreasing them by 42.5% compared to untreated FM rats.

### Melatonin Repressed the Neuroinflammatory Executors in the Spinal Cord of RIFM

The ability of Melatonin to modulate key neuroinflammatory mediators in the FM model was evaluated by measuring the levels of cytokines, specifically TNF-α and IL-1β. These cytokines are critical indicators of neuroinflammation and help to assess Melatonin’s therapeutic potential. In Fig. 5, s.c. Reserpine injection induced neuroinflammation in the rat spinal cord, as evidenced by increased protein levels of (A*) TNF-α (3.53-fold) [F_(2, 15)_ = 139.8, *p* < 0.0001, η^2^ = 0.949], (B*) IL-1β (2.75-fold) [F_(2, 15)_ = 1748, *p* < 0.0001, η^2^ = 0.996], and (C*) IL-6 (2.85-fold) [F_(2, 15)_ = 654.9, *p* < 0.0001, η^2^ = 0.989] compared to naïve control rats (Fig. 5). Melatonin-treated rats attenuated this increase, reducing TNF-α, IL-1β, and IL-6 levels by 47%, 46%, and 47%, respectively, compared to diseased rats.

### Melatonin Inhibited the Up-Regulation of the Spinal Microglial Iba-1 Protein Contents and NMDA Receptor Subunit 2B (NR2B) in the Spinal Cord of RIFM

**Fig. 6:**
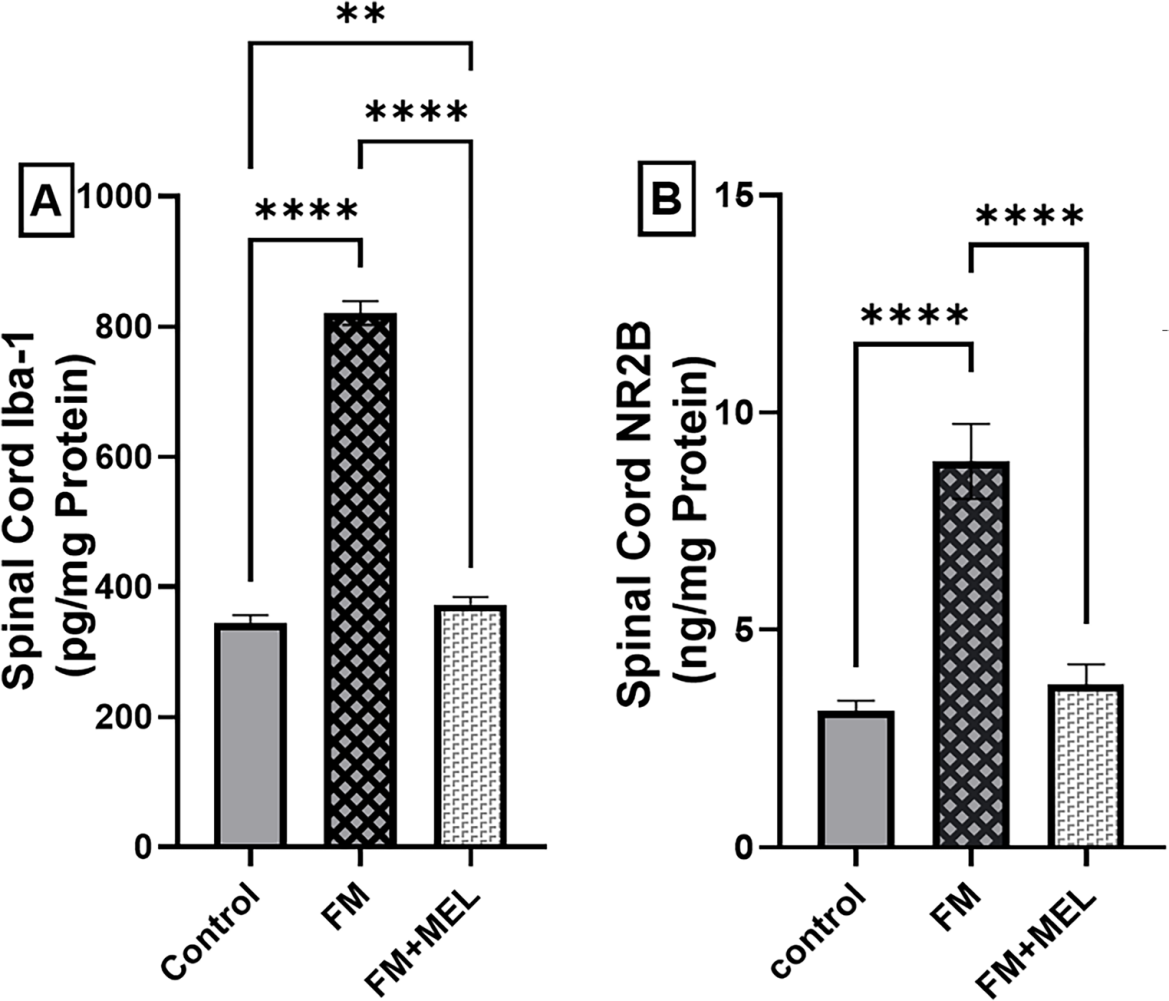
Impact of Melatonin (10 mg/kg, oral, 3 days) on spinal cord (**A**) Iba1 and (**B**) NR2B protein content in the FM-like model. Using one-way ANOVA and Tukey’s post-hoc test (*p < 0.05, **p < 0.01, ***p < 0.001, and ****p < 0.0001), the mean ± SD of rodents (*n* = 6) per group is indicated by each bar with a vertical line. FM: fibromyalgia model, Iba1: ionized calcium-binding adaptor molecule 1, MEL: Melatonin, NR2B: NMDA receptor subunit 2B

To study Melatonin’s efficacy on glutamate dysregulation—identified as a key factor in pain hypersensitivity in the FM model—changes in glutamate receptor NR2B protein content were measured. Additionally, the coincidence between glutamate dysregulation and microglial activation was assessed by evaluating the activated microglial marker Iba-1. An upregulation of Iba1 protein content was detected by ELISA following Reserpine injections, showing a 139% increase [F _(2, 15)_ = 2034, *p* < 0.0001, η^2^ = 0.996] compared to control rats. The Melatonin group significantly reduced SC Iba1 levels to 45% relative to the induction group (Fig. 6 A). As shown in Fig. 6B, reserpinized rats exhibited a 2.83-fold increase in NR2B expression [F _(2, 15)_ = 176.5, *p* < 0.0001, η^2^ = 0.96] compared to the normal group. However, Melatonin-treated rats significantly attenuated this NR2B upregulation by 58% compared to reserpinized rats.

### Melatonin Attenuated the Elevated Levels of Glutamatergic-Related Synaptic Markers: VGLUT, PSD95, NMDA, and AMPA in the Spinal Dorsal Horn in the FM-like Model

**Fig. 7:**
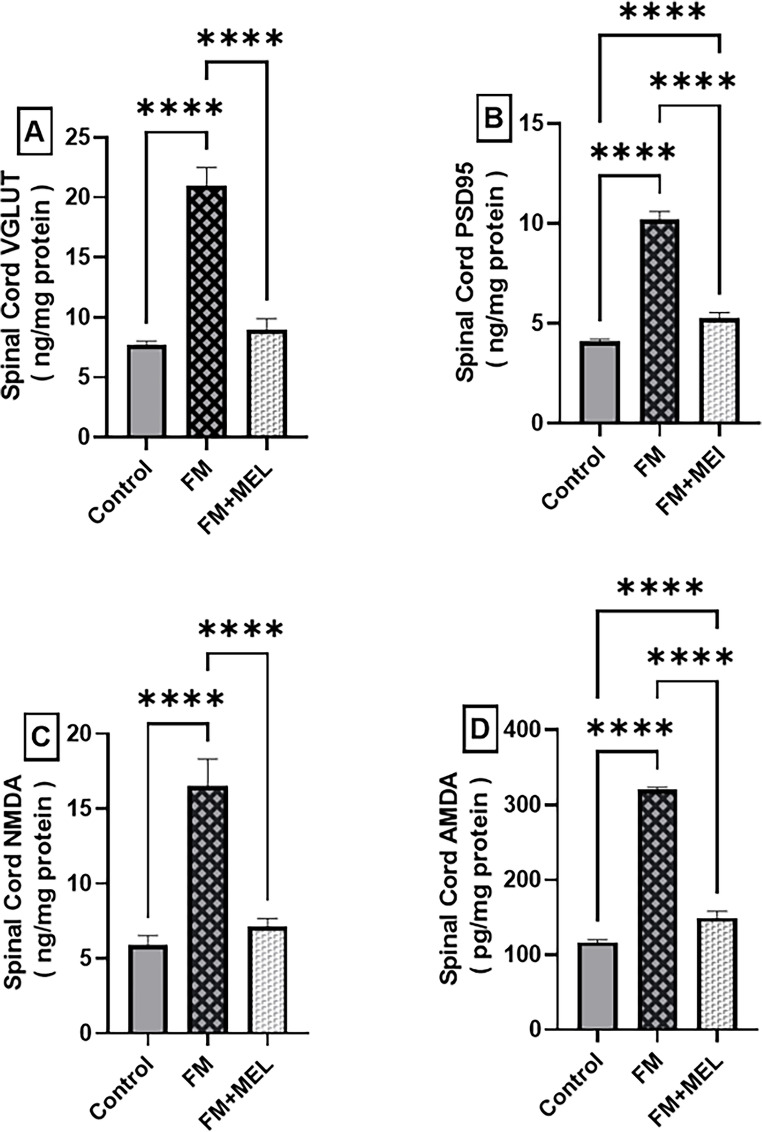
Impact of Melatonin (10 mg/kg, oral, 3 days) on spinal cord (**A**) VGLUT, (**B**) PSD95, (**C**) NMDA, and (**D**) AMPA protein content in the FM-like model. Using one-way ANOVA and Tukey’s post-hoc test (*p < 0.05, **p < 0.01, ***p < 0.001, and ****p < 0.0001), the mean ± SD of rodents (*n* = 6) per group is indicated by each bar with a vertical line. AMPA receptor: α-amino-3-hydroxy-5-methyl-4-isoxazolepropionic acid receptor, FM: Fibromyalgia, MEL: Melatonin, NMDA: N-methyl-D-aspartate receptors, PSD95: Postsynaptic density protein 95, VGLUT: Vesicular glutamate transporter

To determine if Melatonin influences glutamatergic changes in chronic pain, the study evaluated glutamatergic pre- and postsynaptic markers, VGLUT, PSD95, and ionotropic glutamate receptors; NMDA and AMPA in the spinal dorsal horn, a key area for synaptic potentiation and central sensitization in persistent pain. At *p* < 0.0001, the FM group revealed elevated protein levels of all 4 markers, VGLUT, PSD-95, NMDA, and AMPA, reaching 2.7-fold [F(2,15) = 282.4, η² = 0.974], 2.5-fold [F(2,15) = 749.1, η² = 0.991], 2.8-fold [F(2,15) = 156, η² = 0.953], and 4.75-fold [F(2,27) = 35.53], respectively, compared to the naïve rats. Melatonin treatment significantly reduced glutamatergic alterations in the FM spinal dorsal horn. (A) VGLUT and (C) NMDA protein levels were normalized to 43% of control values compared to the untreated group. (B) PSD-95 and (D) AMPA protein levels were also reduced by 48% and 53%, respectively, compared to the FM group after Melatonin treatment (Fig. 7).

### Melatonin Attenuated the Elevated Levels of Glutamatergic-Related Synaptic Markers, PSD-95 and Synaptophysin in the DRG in the FM-like Model

**Fig. 8:**
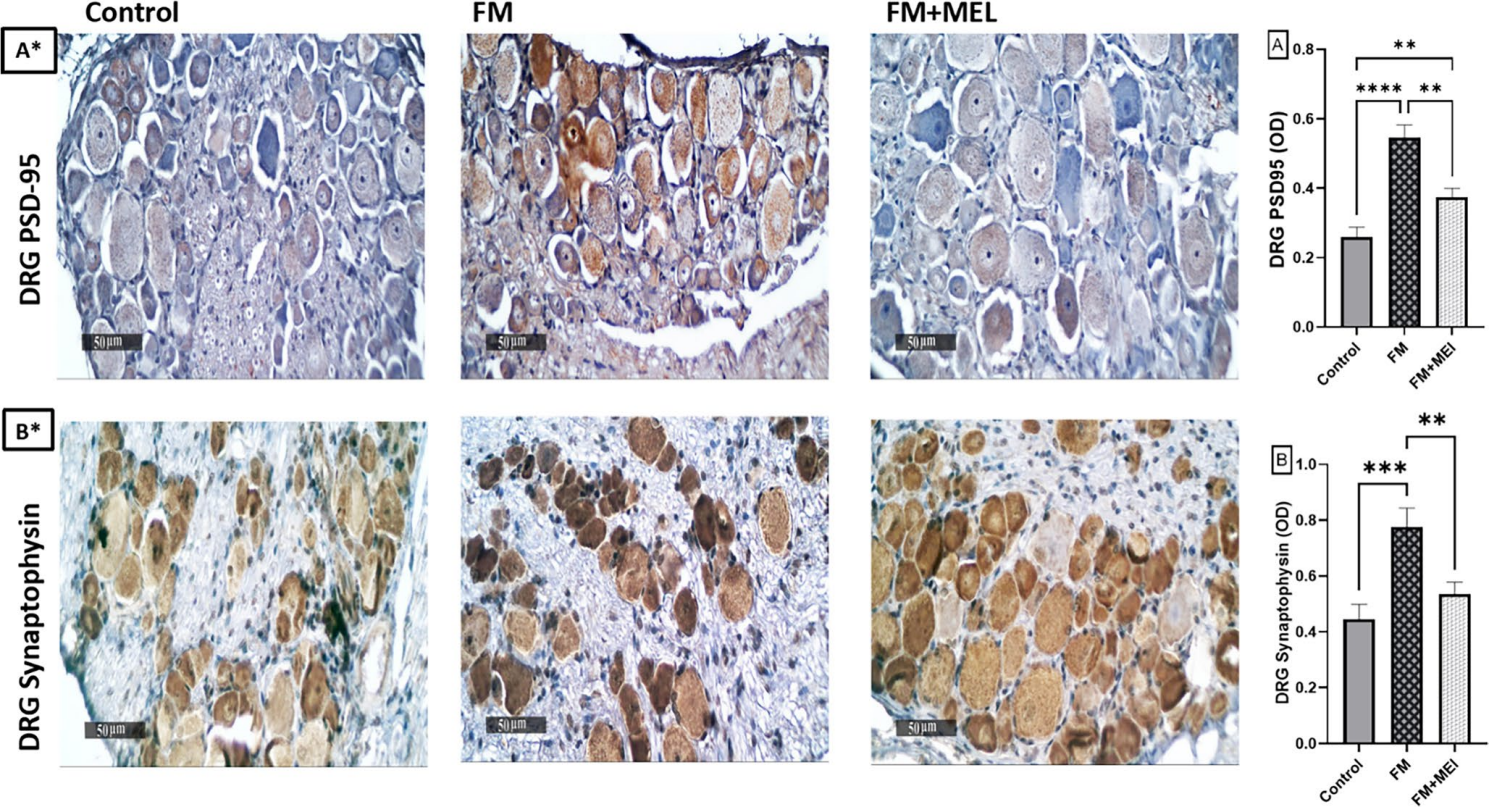
Impact of Melatonin (10 mg/kg, oral, 3 days) on the immunohistochemically expression of (A* & A) PSD-95 and (B* & B) synaptophysin in the DRG of the FM-like model. Panel (A* & B*) shows an increased number of positively stained brown nuclei (black arrow = positive nuclei), indicating elevated PSD-95 and synaptophysin protein expression in reserpinized rats compared to the unstained DRG tissue in the control group. In contrast, Melatonin treatment reduced excitotoxicity, as evidenced by fewer brown-stained nuclei (black arrow = positive nuclei). Each bar in panels A and B with a vertical line represents the mean ± SD of rats (*n* = 3 per group), analyzed using one-way ANOVA followed by Tukey’s post-hoc test (*p < 0.05, **p < 0.01, ***p < 0.001, and ****p < 0.0001). DRG: dorsal root ganglia, FM: fibromyalgia model, MEL: Melatonin, PSD-95: postsynaptic density protein

To study the influence of Melatonin on glutamatergic-related synaptic markers; PSD95 and synaptophysin in the DRG were evaluated. PSD95 is a postsynaptic density protein associated with glutamatergic synapses that assess postsynaptic receptor clustering and synaptic strength. Synaptophysin is a presynaptic vesicle protein that marks synaptic density and activity, including glutamatergic terminals. FM animals exhibited overexpression of PSD-95 and synaptophysin in the DRG compared to normal animals, reaching 2.1-fold [F _(2, 6)_ = 69.16, *p* < 0.0001, η^2^ = 0.96] and 1.75-fold [F _(2, 6)_ = 28.37, *p* = 0.0009, η^2^ = 0.904], respectively. Conversely, Melatonin-treated rats showed a 69% reduction in both PSD-95 and synaptophysin compared to the untreated group (Fig. 8 A and B).

## Discussion

This study demonstrated the antinociceptive role of Melatonin in alleviating pain in a rat model of RIFM, potentially induced by glutamatergic dysregulation in the dorsal horn of the spinal cord. The observations regarding Melatonin’s antinociceptive effect include (i) reduced motor impairments with enhanced mechanical and thermal nociception; (ii) phenotypic improvement, indicated by a minimized rat grimace scale; (iii) amendment of histopathological abnormalities in the DRG; (iv) increased expression of MT1 and MT2 receptors, along with a reduction in glutamate neurotransmission, as indicated by decreased levels of PSD95 and synaptophysin in DRG and VGLUT, PSD95, NMDA, and AMAPA levels in spinal cord; (v) mitigation of mitochondrial dysfunction through activation of the SIRT1/PGC-1α pathway; (vi) alleviation of neuroinflammation, as evidenced by reduced microglial activation, recognized by reduced Iba-1 levels and accompanied by decreased levels of pro-inflammatory factors such as IL-1β, IL-6, and TNFα; and (vii) low expression of pro-inflammatory cytokine regulators, including p38 MAPK and NF-κB. Notably, this study highlights the significance of Melatonin-mediated antinociceptive action in repairing mitochondrial dysfunction, glutamatergic dysregulation, and microglial activation, all of which contribute to the disruption of the ascending pain pathway in the DRG of the FM rat model. Concomitantly in the present study, RIFM rats exhibited heightened pain sensitivity, neuromuscular disability, and motor incoordination, as indicated by their performance in the neurobehavioral assessments.

Reserpine significantly decreased pain threshold in the von Frey test, Randall-Sellito test, cold allodynia, and hot plate tests. Additionally, supraspinal pain was increased, as noted in the tail suspension test. These outcomes are consistent with previous research findings (Brum et al. 2020, 2022). De la Luz-Cuellar et al. (2019) demonstrated that mechanical allodynia and muscle hyperalgesia peak on the 7th day following Reserpine administration, with symptoms gradually subsiding by day ten (De la Luz-Cuellar et al. 2019). In the present study, Melatonin was started on day 7 and continued for three consecutive days, prompting consideration of whether the observed effects are attributable to Melatonin or influenced by the spontaneous resolution of Reserpine-induced nociception. The current findings revealed that mechanical and thermal hypersensitivity persisted in the Reserpine-only group after day seven, confirming that the pronociceptive effects of Reserpine remained active at the initiation of Melatonin. This persistence strongly supports the conclusion that the attenuation of pain behaviors in the Melatonin-treated group results from Melatonin’s pharmacological efficacy rather than spontaneous recovery. Moreover, prior investigations on RIFM have established a prolonged pain state lasting a minimum of 14 days and in some cases extending up to 21 days post-administration (Fusco et al. 2019; Yao et al. 2020; Mohamed et al. 2025; Shafiek et al. 2025a, b; Kamaly et al. 2025). Additionally, Melatonin, and its agonists and antagonists were administered intrathecally before or after intradermal capsaicin injection, where Melatonin and its agonists significantly decreased mechanical allodynia and hyperalgesia. In contrast, Melatonin antagonism increased the pain withdrawal frequency (Tu et al. 2004). Altogether, these findings revealed that activation of the endogenous Melatonin system in the spinal cord can mitigate central sensitization in FM. These data validate the therapeutic potential of Melatonin in mitigating sustained nociceptive sensitization.

Regarding motor performance in the Rota rod, Reserpine significantly impaired Rota rod performance, simulating motor deficits observed in FM. This finding aligns with previous studies reporting neurobehavioral impairments following Reserpine administration (Yao et al. 2020; Atta et al. 2023b; Mohamed et al. 2025; Kamaly et al. 2025). However, some studies have recorded no significant changes or milder deficits in Rota Rod performance, reflecting differences in experimental protocols, such as training frequency. In the present study, the three training sessions may have pronounced fatigue effects, while another study with fewer training sessions (Zhang et al. 2016) reported less notable deficits. Melatonin demonstrated its potential to improve motor performance in various models, including stroke (Zhao et al. 2024), Parkinson’s disease (Rasheed et al. 2018), peripheral neuropathy (El-Sawaf et al. 2024), and cerebral ischemia-reperfusion injury (Yilmaz et al. 2023), particularly at lower doses not exceeding 20 mg/kg. Some studies indicate that higher doses (120–150 mg/kg) of Melatonin may impair Rota rod performance or show limited efficacy, showing the importance of dose consideration (Arreola-Espino et al. 2007; Çakirgöz et al. 2025). Additionally, delayed effects of Melatonin on motor impairment have been reported in models rather than FM, where motor recovery was noted after 7 days of treatment despite no improvement after 3 days (Yilmaz et al. 2023). These discrepancies may be attributed to differences in pathology, treatment duration, and timing across models.

Abnormal pain sensitization is attributed to excessive glutamatergic neurotransmission in the spinal dorsal horn of FM. Contrary to the normal state, glutamate receptors involved in nociceptive transmission, such as NMDARs, are over activated due to increased glutamate release from dorsal horn terminals, which enhances spinal wind-up and hyperalgesia (Staud and Domingo 2001; Pereira and Goudet 2019). Ferrari et al. (2014) demonstrated that intrathecal administration of glutamate increased the central nociceptive reflex excitability in the DRG. In contrast, intrathecal administration of NMDA receptor antagonists reversed this milieu in experimental chronic pain models (Zhou et al. 2011; Ferrari et al. 2014). Unfortunately, NMDAR antagonists produce inconsistent results or cause severe side effects. This underscores the necessity to develop new therapies targeting glutamatergic dysregulation to alleviate pain hypersensitivity without blocking glutamate receptors (Temmermand et al. 2022).

Consistent with the previously mentioned pain-exacerbating mechanism, this study investigated the glutamatergic alteration in the dorsal horn spinal cord and in the DRG. The present results demonstrated a significant upregulation of VGLUT, PSD95, NMDA, and AMPA receptor expression within the dorsal horn. These alterations indicate enhanced glutamatergic neurotransmission and synaptic strengthening, which are hallmark features of central sensitization. The concurrent increase in both presynaptic VGLUT and postsynaptic PSD95, NMDA, and AMPA markers suggests an overall facilitation of excitatory synaptic drive, further supporting the transition of pain. Similar observations have been reported in various chronic pain models, where spinal glutamatergic overactivity plays a critical role in maintaining persistent pain states (Liu and Salter 2010; Niciu et al. 2012; Bardoni 2013; Turan Yücel et al. 2023; Jang and Garraway 2024).

The synaptic scaffolding molecule, PSD-95, binds to the NMDA 2B subunit (NR2B). The binding of NR2B to PSD-95 contributes to dorsal horn neuron hyperexcitability and elevated pain-associated behaviors, such as hyperalgesia and allodynia, in human and animal models. Subsequently, NR2B activation is involved in promoting central sensitization and nociplastic pain (d’Mello et al. 2011; Li et al. 2022). In addition to the dorsal horn, PSD-95 upregulation in the DRG further supports the occurrence of widespread synaptic plasticity contributing to the maintenance of chronic pain. The present model corroborates previous findings of synapse-like remodeling around DRG somata in chronic pain and synaptic marker enrichment in human DRG neurons revealed through increased PSD95 and synaptophysin levels in the DRG (Sun et al. 2006; Cheng et al. 2015; Yu et al. 2024). The expression levels of PSD-95 and synaptophysin in rat DRG as each is linked to glutamatergic neurotransmission in the nervous system. The current study showed a remarkable increase in synaptophysin levels in the DRG after RES injection in rats. These findings support that the dorsal horn of the spinal cord and the DRG glutamatergic system regulate hyperalgesia in FM.

Melatonin demonstrated neuroprotective, anti-inflammatory, and anti-apoptotic efficacy. Herein, the novelty lies in revealing Melatonin’s potential to counteract excessive glutamatergic dysregulation and the nociplastic state in FM. This was confirmed by immunohistological analysis, which showed that DRG tissues in the Melatonin-treated group exhibited lower PSD-95 and synaptophysin expression, as well as decreased NR2B expression in the spinal cord. This reflects Melatonin’s ability to amend the exaggerated glutamatergic transmission observed in FM, thereby alleviating pain transmission, as seen in behavioral tests. These findings align with a previous clinical study on Melatonin’s impact on the pain modulatory system in female FM patients. Which demonstrated that Melatonin enhanced pain reduction, as reflected by improvements in the outcomes of the visual analog scale, heat and pressure pain thresholds, FM impact questionnaire, and numerical rating pain scale (de Zanette et al. 2014).

Additionally, preclinical studies indicated that the injection of Melatonin significantly suppresses or completely eliminates wind-up activity in the spinal cord of rats. This suppressive action is likely associated with Melatonin’s agonistic properties at its receptors in dorsal horn neurons (Laurido et al. 2002; Noseda et al. 2004). However, the molecular mechanism by which Melatonin exerts its neuroprotective action remains unclear. MT1 and MT2 are two distinct families of membrane receptors located in the plasma membrane, both of which are abundantly expressed in the CNS. Both receptors have been identified in the dorsal horn of the spinal cord, particularly in laminae I-V and X, which are involved in pain regulation mechanisms (Srinivasan et al. 2012; Das et al. 2013). The present research emphasizes their role in the FM experimental model, as their expression levels were inversely proportional to glutamate and pain hypersensitivity. This is consistent with Das et al. (2013); the study confirmed the contribution of MT1/2 in protecting neurons from glutamatergic dysregulation (Das et al. 2013). The knockdown of endogenous MEL receptors was performed using small interfering RNA to elucidate the receptor-dependent neuroprotective efficacy of Melatonin. Results from the silenced endogenous MT1 and MT2 receptors support the function of Melatonin receptors in modulating cellular responses to excitotoxic injury (Das et al. 2013).

The apparent discrepancy between Melatonin’s agonist activity and the observed upregulation of MT1 and MT2 mRNA in the present study can be explained by the context-dependent regulatory dynamics of Melatonin receptors in pathological states. In neurodegenerative amyotrophic lateral sclerosis and Huntington’s disease, as well as age-related pathologies, MT1/MT2 receptors are frequently downregulated due to oxidative stress and mitochondrial dysfunction. Melatonin prevented the disease-associated decline in MT1 protein in spinal motor neurons, as confirmed by immunostaining and mRNA analyses (Wang et al. 2011; Zhang et al. 2013; Jenwitheesuk et al. 2017; Romano et al. 2024). In the context of FM, a condition linked to oxidative stress and mitochondrial dysfunction, the observed mRNA was downregulated in the disease and reversed by Melatonin administration. This restoration may be owed to re-establishing homeostatic receptor expression in pathological statuses.

Besides glutamatergic dysregulation, mitochondrial dysfunction and neuroinflammation were investigated as potential causes of FM. Maintaining mitochondrial function is critical for managing sensory and chronic pain (Tu et al. 2004; Flatters 2015). However, few existing studies provide insight into the relationship between Melatonin receptors and mitochondrial biogenesis in DRG. The results revealed that Melatonin efficiently alleviated pain hypersensitivity in animal models by boosting SIRT1 and its substrate, PGC-1α. These results align with in vitro data suggesting that paclitaxel disrupts mitochondrial membrane potential and metabolic activity in DRG cells, which are subsequently restored by Melatonin (Galley et al. 2017). Melatonin can traverse cell membranes and accumulate inside mitochondria, interacting with mitochondrial MT1 receptors to regulate mitochondrial function. Moreover, Melatonin preferentially accumulates in mitochondria and counteracts mitochondrial-derived ROS by binding to MT1 and MT2, resulting in analgesic effects in several neuropathic pain models (Zeng et al. 2023).

Recent research found that the aberration of the SIRT1/PGC-1α signaling pathway is involved in the development of neuropathic pain. SIRT1 knockdown in naïve rats resulted in pain behavior (Ling-Jun Xu et al. 2023), while intrathecal administration of the SIRT1 activator SRT1720 drastically decreased chronic constriction injury-induced allodynia (Lv et al. 2015). Notably, SIRT1/PGC-1α levels were significantly decreased in the spinal cord of a rat model, causing mitochondrial dysfunction and overproduction of pro-inflammatory cytokines, including IL-1β and TNF-α, which sensitize neurons and induce neuropathic pain (Zeng et al. 2023). Furthermore, SIRT1-mediated deacetylation of PGC-1α mitigated glutamatergic dysregulation in cortical neurons (Jia et al. 2016). Zeng et al. (2023) highlighted Melatonin’s ability, via MT2, to improve mitochondrial function and mitigate neuropathic pain through the SIRT1/PGC-1α pathway in the DRG of rats (Zeng et al. 2023). Similarly, the present study demonstrated that Melatonin injection promotes SIRT1, which activates PGC-1α through deacetylation. Previously, Melatonin showed comparable therapeutic effects against FM by targeting key pathological mechanisms, including oxidative stress, inflammation, mitochondrial dysfunction in muscle tissue, and neuroimmune activation in the brain. It has been shown to alleviate motor impairments and improve musculoskeletal structure by reducing inflammation, mitochondrial dysfunction, and oxidative stress markers in the gastrocnemius muscle, indicating its potential in managing FM-related musculoskeletal damage (Favero et al. 2017, 2019). This explains part of the antinociceptive effect of Melatonin, particularly its role in restoring mitochondrial function and alleviating glutamatergic dysregulation, as investigated in RIFM.

Interestingly, earlier research indicated that microglial activation in the spinal cord contributes to neuroinflammation and chronic pain conditions like FM by influencing glutamate release. This process involves the activation of microglia to the M1 phenotype in the dorsal horn, triggering the release of pro-inflammatory cytokines, including TNFα and IL-1β/−6, as well as glutamate, leading to neuroinflammation, synaptic hyperexcitability, and central sensitization. The present study examined microglial status in the spinal cord, where Iba1 immunoreactivity was markedly elevated in activated microglia, consistent with previous findings (Ito et al. 1998; Jung et al. 2020). Conversely, Melatonin significantly reduced Iba1 expression levels, potentially through the NF-κB and MAPK pathways, which have been identified as key regulators of central sensitization besides the role of pro-inflammatory cytokines, IL-1β, and TNF-α (Fusco et al. 2019). This was evident from inhibiting the p38/MAPK pathway, which restricted microglial activation and neuroinflammation, subsequently alleviating allodynia and hyperalgesia (Bennett 2005). The current results and those of Bennett (2005) reinforce the potential of p38 MAPK pathway inhibitors as a novel approach to pain management in FM patients (Bennett 2005). In the present study, Melatonin-treated rats exhibited downregulation of NF-κB and p38 MAPK, which may be partially attributed to mitochondrial restoration. PGC-1α activation has been demonstrated to inhibit NF-κB signaling in the brain, thereby preventing the release of pro-inflammatory molecules such as TNFα. This promotes microglial activation, with further research needed to elucidate its activation (Yang et al. 2017; Qin et al. 2018; Castro et al. 2022), as is well observed here in Melatonin-treated animals.

The primary limitation of this study is the lack of investigation into the distinct roles of MT1 and MT2 receptors in modulating neuroinflammation and pain signaling within the spinal cord and DRG in the FM animal model. Additionally, the effects of Melatonin on descending pain modulatory pathways, including serotonergic and noradrenergic neurotransmission, were not explored. Body weights were also not monitored throughout the entire experimental period, and assessing Iba1 alone did not clarify the microglial phenotypes. Addressing these gaps is essential for a comprehensive understanding of receptor-specific mechanisms underlying Melatonin’s analgesic and anti-inflammatory effects.

### Compliance with ethical standards

PT 3478 is the approval number assigned to this investigation by the Animal Care and Use Ethics Committee of the Faculty of Pharmacy, Cairo University. The study was conducted in accordance with the ARRIVE 2020 standards and the guidelines specified in the Guide for the Care and Use of Laboratory Animals, which was published by the US National Institutes of Health (NIH publication No. 85–23, revised 2011). The study’s animals were subjected to all feasible safeguards to mitigate their distress.

### Induction of the FM-like Model

In the neck region, RES (Sigma-Aldrich, Saint Louis, MO, USA) was administered subcutaneously (s.c.) at a dose of 1 mg/kg/day for three days. The preparation was a 0.5% glacial acetic acid solution (Nagakura et al. 2009).

### Experimental Design

**Scheme 1:**
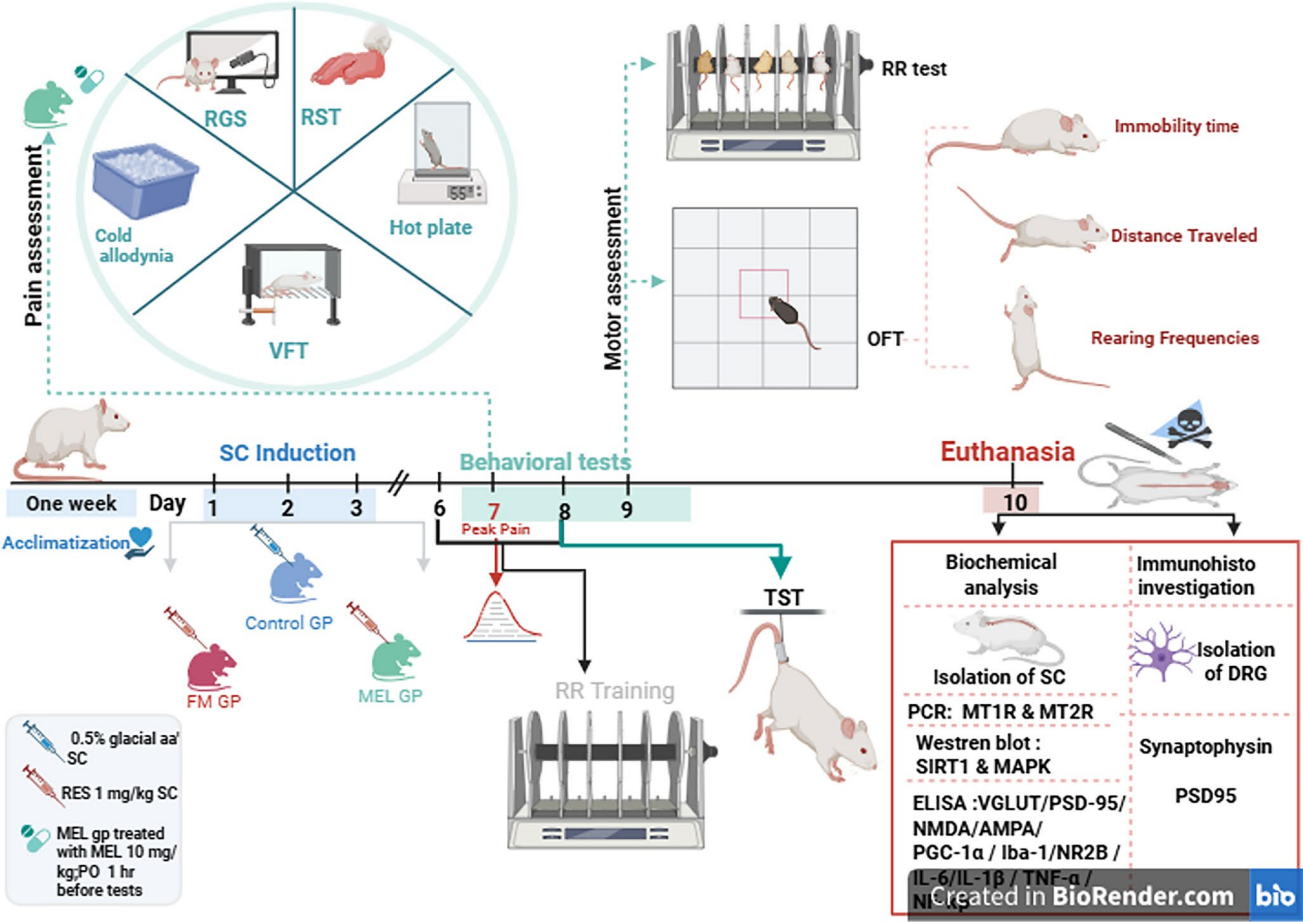
Experimental study diagram. AMPA receptor: α-amino-3-hydroxy-5-methyl-4-isoxazolepropionic acid receptor; DRG: dorsal root ganglia; ELISA: enzyme-linked immunosorbent assay; FM GP: fibromyalgia group; Iba-1: binding adaptor molecule 1; IL-1β: interleukin-1 beta; IL-6: interleukin-6; MAPK: mitogen-activated protein kinases; MEL GP: Melatonin group; MT1R: Melatonin receptor 1; MT2R: Melatonin receptor 2; NF-κB: nuclear factor kappa B; NMDA: N-methyl-D-aspartate receptors; NR2B: NMDA receptor subunit 2B; OFT: open field test; PCR: polymerase chain reaction; PGC-1α: peroxisome proliferator-activated receptor gamma coactivator 1-alpha; p.o.: per oral; PSD95: postsynaptic density protein 95; RES: Reserpine; RGS: rat grimace scale; RR: rotarod; RST: Randall-Selitto mechanical threshold; s.c.: subcutaneous; SIRT1: silent information regulator sirtuin 1; TNF-α: tumor necrosis factor-alpha; TST: tail suspension test; VGLUT: vesicular glutamate transporter; VFT: von Frey test

In Scheme 1, the animals were randomly assigned to three groups, each consisting of ten rats. In order to dispel allocation bias, a random number generator was implemented. With a power of 0.8, an effect size of 0.6, and an alpha level of 0.05, the G*Power calculator version 3.1 (Düsseldorf, Germany) was employed to determine the sample size.

Group I served as the control group, receiving distilled water with 0.5% glacial acetic acid (s.c.) for three days, followed by distilled water with 1% DMSO. Group II, designated as the FM group, received RES (1 mg/kg, s.c.; Sigma-Aldrich, MO, USA) for three days. Group III (FM + Melatonin) received RES (1 mg/kg, s.c.) for three days, followed by Melatonin (10 mg/kg, p.o.; Sigma-Aldrich, MO, USA) dissolved in 1% DMSO in distilled water, administered for three days starting on day 7, one hour before behavioral testing (Galley et al. 2017). Melatonin’s dose was selected based on extensive preclinical evidence demonstrating its efficacy in alleviating pain without inducing motor or sedative side effects (Galley et al. 2017; Tancheva et al. 2021). On the seventh day, the pain reached its peak, consistent with previous findings (De la Luz-Cuellar et al. 2019; Ikeda et al. 2023). The Grimace scale was used to assess spontaneous pain, while mechanical and thermal sensitivities were evaluated using Von Frey, Randall-Sellito, hind paw cold allodynia, and hot plate tests. The next day, the TST was performed to assess supraspinal pain. On the ninth day, behavioral tests examining motor activity, muscle fatigue, and coordination were conducted using the open field (OFT) and rotarod (RR) tests. Motor behavior was subsequently recorded using the ANY-maze video tracking software (Stoelting Co., Illinois, USA). Researchers were blinded to group assignments during behavioral assessments and data analysis to minimize observer bias. A two-hour resting period was implemented between tests, beginning with the least stressful and concluding with the most stressful, to mitigate animal suffering. The testing sequence was chosen to avoid prior mild thermal stimulation that can sensitize animals to subsequent mechanical stimuli (Gröne et al. 2012). All instruments were sanitized with 70% ethanol after each experiment. Behavioral evaluations were conducted according to the established norms and standard practices of the Neurobehavioral Core Lab (Faculty of Pharmacy, Cairo University). All data collectors and outcome assessors remained blinded to treatment groups until data analysis was completed.

### Tissue Collection

Animals were euthanized through decapitation under pentobarbital anesthetic (200 mg/kg, i.p.) after the completion of all behavioral evaluations (Zatroch et al. 2016). The spinal cords were individually isolated, cleansed with saline, and weighed. Spinal cords were divided into two subgroups. The first subgroup (*n* = 3) was preserved in 10% neutral buffered formalin and utilized for histological and immunohistochemical analysis of DRG. The second subgroup from the remaining rats’ spinal cords were excised, swiftly frozen in liquid nitrogen, and stored at − 80 °C. To ensure consistent analysis, each spinal cord was divided rostro-caudally into two equal halves (upper and lower segments). The lower half of the study (*n* = 6) was utilized to VGLUT, PSD95, NMDA, AMPA, PGC1α, NF-κB, Iba-1, TNF-α, IL-6/−1β, and NR2B using the ELISA technique. The upper half of the samples was used to evaluate MT1R and MT2R via quantitative real-time polymerase chain reaction (qPCR) (*n* = 3), and SIRT-1 and MAPK were assessed using western blot (*n* = 3). The remaining spinal cord tissue was stored at −80 °C for possible further validation analyses. In Scheme 1, the experimental design is illustrated.

isoxazolepropionic acid receptor; DRG: dorsal root ganglia; ELISA: enzyme-linked immunosorbent assay; FM GP: fibromyalgia group; Iba-1: binding adaptor molecule 1; IL-1β: interleukin-1 beta; IL-6: interleukin-6; MAPK: mitogen-activated protein kinases; MEL GP: Melatonin group; MT1R: Melatonin receptor 1; MT2R: Melatonin receptor 2; NF-κB: nuclear factor kappa B; NMDA: N-methyl-D-aspartate receptors; NR2B: NMDA receptor subunit 2B; OFT: open field test; PCR: polymerase chain reaction; PGC-1α: peroxisome proliferator-activated receptor gamma coactivator 1-alpha; p.o.: per oral; PSD95: postsynaptic density protein 95; RES: Reserpine; RGS: rat grimace scale; RR: rotarod; RST: Randall-Selitto mechanical threshold; s.c.: subcutaneous; SIRT1: silent information regulator sirtuin 1; TNF-α: tumor necrosis factor-alpha; TST: tail suspension test; VGLUT: vesicular glutamate transporter; VFT: von Frey test.

### Neurobehavioral Tests

#### Rat Grimace Scale

A video camera was used to assess spontaneous pain in rats by capturing images of their facial expressions every 3 min, resulting in 10 photographs over 30 min. The photographs were then randomly organized in PowerPoint for evaluation on a 3-point scale. They were assessed based on four distinct action units: orbital tightness (eye), ear alteration, nose/cheek flattening, and whisker modification. Each action unit was scored on a 3-point scale (0 = nonexistent, 1 = moderately visible, 2 = definitely present) (Nagakura et al. 2019; Tanei et al. 2020).

#### Open Field Test (OFT)

The open field test (OFT) was employed to evaluate the spontaneous locomotor activity of animals. A black box measuring 80 cm by 80 cm and 40 cm in height was used for this test. Each rat was positioned in the center of the box and allowed to wander autonomously for three minutes. The behavior of the rat was captured by a video camera that was positioned above the box. The camera recorded the total distance traveled in meters (OFT DT), immobility time (the extent of lack of movement during testing, OFT IT), and the frequency of horizontal locomotor and vertical activity, known as rearing (OFT RF). Cleaning the apparatus with 70% ethanol following each animal trial and allowing it to dry entirely before the next trial was conducted to prevent any potential bias from aroma (Ibrahim et al. 2024).

#### Von Frey Test (VFT)

The VFT uses specialized filaments to determine the mechanical allodynia thresholds in rats. A manual protocol was employed to assess the mechanical sensitivity of the animals. After a 30-minute acclimatization period on a raised mesh floor in an acrylic cage, the rats’ hind legs were stimulated five times using calibrated von-Frey filaments. The minimal force-inducing withdrawal response was established as the mechanical threshold, and a positive response was recorded. A response was considered positive if the animal displayed any nociceptive activity, such as rapid paw withdrawal, licking, or shaking of the paw, either during stimulus administration or immediately following filament removal, and was documented as paw withdrawal threshold (Deuis et al. 2017; Ikeda et al. 2023).

#### Rotarod (RR) Test

The RR was deployed to assess fatigue-like symptoms in rats administered RES to induce FM. Before the experimental procedures, the rats underwent training for three consecutive days, with each session lasting one minute, on an automated five-lane RR device (Model 47750, Ugo Basile, Italy) that accelerated from 4 to 20 rpm. On the fourth day, a fatigue analysis was conducted at 20 rpm, and the time the rats remained on the rod was documented as rotarod falling latency (RR FL) (Dagnino et al. 2019).

#### Randall-Selitto Test (RST)

To assess mechanical hyperalgesia, the RST was implemented. The analgesiometer (Model 7200, Ugo Basile, Italy) was employed to evaluate the withdrawal threshold by applying a continuous mechanical force to the mid-gastrocnemius muscle of the rat’s rear limb. The Randall-Sellito mechanical threshold (RS MT) was determined by recording the hind limb withdrawal of rats under restraint with a soft cotton fabric. In an effort to guard against tissue injury, the threshold burden was restricted to 250 g (Kamaly et al. 2025).

#### Hind Paw Cold Allodynia Test

The hind paw cold allodynia test is employed to assess cold thermal sensitivity by assessing the withdrawal response of the rat’s hind paw when it is delicately immersed in ice-cold water at 4 ± 1 °C. We assessed cold allodynia using paw withdrawal latency (CAPWL), with a threshold duration of 20 s (Ibrahim et al. 2024).

#### Hot Plate Test

The hot plate test is a commonly used technique for evaluating supraspinal thermal nociception in rats and is often employed to detect thermal hyperalgesia. In this experiment, rats are positioned on a confined hot plate maintained at a temperature of 55 ± 1 °C, and the time taken to lick a hind paw or escape the enclosure is designated as hot plate reaction latency (HP RL), indicating the threshold of thermal pain response in rats. A cutoff reaction time of 20 s is used to prevent physical injury (Ibrahim et al. 2024; Kamaly et al. 2025).

#### Tail-suspension Test (TST)

The tail suspension test is based on the fact that a rat, when hung by its tail, displays alternating phases of agitation and stillness. In this experiment, animals were elevated 50 cm off the ground using adhesive tape, with their forelimbs stabilized by two smooth, V-shaped slopes to prevent excessive agitation. The duration of immobility was quantified during a 6-minute trial and recorded as the tail suspension test immobility time (TST IM) (Ibrahim et al. 2024).

### Histopathological Examination

Rat dorsal root tissue samples from lumbar segments were preserved in 10% neutral buffered formalin for 72 h and subsequently subjected to a series of ethanol grades, cleaned with xylene, and then infiltrated and embedded in Paraplast tissue embedding medium (Leica Biosystems). Serial slices of dorsal root ganglion tissue, each five micrometers thick, were prepared using a rotary microtome for demonstration. Tissue slices were stained with hematoxylin and eosin (H&E) using the standard staining process for blinded light microscopic inspection conducted by an expert histologist. Culling (2013) states that all normal protocols for sample processing were followed (Culling 2013).

### Immunohistochemical Examination

In accordance with the manufacturer’s protocol, immunohistochemistry staining was performed on paraffin-embedded DRG tissue slices that were 5 microns thick. 0.3% H2O2 was applied to deparaffinized dorsal root ganglion tissue segments for a duration of 20 min. The DRG was incubated at 4 °C overnight with an anti-PSD95 antibody (Abcam, ab192757, 1:200) and an anti-synaptophysin antibody (Abcam, ab14692, 1:100). The HRP Envision kit secondary antibody (Cat.#K5007) was incubated with the tissue sections for 20 min after they were rinsed with PBS. The specimens were subsequently cleansed and subjected to diaminobenzidine (DAB) treatment for 15 min. Subsequently, they were washed with PBS, counterstained with hematoxylin, dehydrated, cleared in xylene, and mounted for microscopic analysis. The mean PSD95 and synaptophysin optical densities in immunohistochemically stained sections were determined by randomly selecting and examining a minimum of six non-overlapping fields. The mean of these 6 values was averaged for each animal, and then statistical analyses were performed on these per-animal means as independent data points. The histologist utilized the Leica application module for histological analysis, which was integrated with a Full HD microscopic imaging system (Leica Microsystems GmbH, Germany) (Culling 2013).

### Biochemical Parameters

#### Enzyme-linked Immunosorbent Assay (ELISA)

Rat-specific ELISA kits were deployed to ascertain the following parameters in the tissue samples of the SC: PGC1α (Cat.#MBS1600213), Iba-1 (Cat.#MBS3809193), NF-κB (Cat.#MBS2505513), and IL-1β (Cat.#MBS825017; all from My BioSource, San Diego, CA, USA). Cusabio ELISA kits were used to evaluate TNF-α (Cat.#CSB-E11987r, Houston, TX, USA). In addition, ELISA kits from R&D Systems were used to quantify IL-6 (Cat.#R6000B, Minneapolis, MN, USA). Moreover, N-methyl-D-aspartate receptor subunit 2B (NR2B) (AFG Bioscience, Cat. No. EK721705, Northbrook, IL, USA), VGLUT (My BioSource, San Diego, CA, USA; Cat.#MBS2611774), PSD95 (Sandwich ELISA; LS-F7142, LSBio, Seattle, WA, USA), NMDA (My BioSource, San Diego, CA, USA; Cat.#MBS269995), and AMPA (ELK Biotechnology, Cat.# ELK6368, Wuhan, Hubei, China) were assessed according to the manufacturer’s instructions. AMPA, TNF-α, NF-κB, and IL-6/IL-1β were quantified in pg/mg protein, while VGLUT, PSD95, NMDA, PGC1α and NR2B were measured in ng/mg protein. Protein concentrations were assessed using a BCA kit from G-Bioscience (USA), employing a standard curve to convert measurements to pg/ml or ng/ml. These values were normalized by dividing by the tissue protein concentration (mg/ml), yielding expressions in pg/mg or ng/mg protein.

#### Western Blot of P38 MAPK and SIRT1

Following the quantification of protein levels with the BCA test kit (Bio-Rad, CA, USA), the spinal cord’s SIRT1 and MAPK protein expressions were evaluated. Using SDS-PAGE, equal portions of protein samples were separated and subsequently deposited onto a nitrocellulose membrane. Subsequently, the membranes were incubated at 4 °C overnight with primary antibodies that were specific to SIRT1 (Cat.#PA5-116530) and phospho-p38 MAPK (Cat.#36–8500), sourced from Thermo Fisher Scientific, MA, USA. Later, an appropriate horseradish peroxidase-conjugated secondary antibody (Dako, Glostrup, Denmark) was applied and incubated overnight. Following this, the bands were visualized using the Western Lightning Plus ECL chemiluminescence reagent (Perkin Elmer, MA, USA), which was captured using a Chemi-Doc imager (Bio-Rad, CA, USA). The optical density of the findings was standardized to β-actin (Cat.#PA1-183) for SIRT1 and total phospho-p38 MAPK (Cat.#AHO1202), both from Thermo Fisher Scientific, MA, USA.

#### qPCR Experiment

Utilizing the 2^−ΔΔCT^ equation, the relative expression of spinal MT1R and MT2R mRNA in relation to β-actin was determined. Utilizing the SV total RNA isolation system (Cat.#Z3101; Promega, WI, USA), total RNA was extracted and subsequently transformed to cDNA using an Invitrogen kit (CA, USA). SYBR Green PCR master mix (Cat. #4309155; Applied Biosystems, CA, USA) was employed to run the qPCR. Table 1 contains primer sequences for the parameters that were examined.

**Table 1:** Primer sequences

Parameters	Primer sequences
MT1R	F:5-CGGACAGCAAACCCAAACTG-3 R:5-AACTAGCCACGAAGAGCCAC-3
MT2R	F:5-CATCTGTCACAGTGCGACCT-3 R:5-TGCTGGCTGTCTGGATGAAG-3
Β-actin	F:5-CCCGCGAGTACAACCTTCTT-3 R:5-AACACAGCCTGGATGGCTAC-3

### Statistical analysis

Mean ± standard deviation was used for representing the data, and significance was established at *p < 0.05, **p < 0.01, ***p < 0.001, and ****p < 0.0001. The means were compared using a one-way analysis of variance (ANOVA) and Tukey’s multiple-comparison test in GraphPad Prism (version 9; San Diego, CA, USA). Statistics were conducted to analyze the data. The effect size, partial eta squared (η²), statistical significance (p), degrees of freedom (df), and F-value (F) were specified for each effect.

## Conclusion

In conclusion, this study demonstrates that Melatonin exerts significant antinociceptive and anti-inflammatory effects in a Reserpine-induced FM model. Melatonin effectively alleviates nociplastic pain and associated behavioral disturbances by modulating glutamatergic-related synaptic markers in the DRG, restoring mitochondrial function, and reducing microglial activation in the spinal cord. These findings support the potential therapeutic role of Melatonin in managing FM symptoms and highlight its capacity to target molecular abnormalities in pain pathways.

## Supplementary Information

Below is the link to the electronic supplementary material.

Supplementary Material 1 (JPG.926 KB)

Supplementary Material 2 (JPG.1.08 MB)

Supplementary Material 3 (JPG.1.17 MB)

Supplementary Material 4 (JPG.1.27 MB)

## Data Availability

All relevant data are present in the submitted manuscript, table, and figures. Any other data will be made available on request.

## References

[CR1] Arreola-Espino R, Urquiza-Marín H, Ambriz-Tututi M et al (2007) Melatonin reduces formalin-induced nociception and tactile allodynia in diabetic rats. Eur J Pharmacol 577:203–210. 10.1016/j.ejphar.2007.09.006 17920585 10.1016/j.ejphar.2007.09.006

[CR2] Atta AA, Ibrahim WW, Mohamed AF, Abdelkader NF (2023a) Microglia polarization in nociplastic pain: mechanisms and perspectives. Inflammopharmacology 31:1053–1067. 10.1016/j.ejphar.2023.175810 37069462 10.1007/s10787-023-01216-x PMC10229465

[CR3] Atta AA, Ibrahim WW, Mohamed AF, Abdelkader NF (2023b) Targeting α7-nAChR by galantamine mitigates reserpine-induced fibromyalgia-like symptoms in rats: involvement of cAMP/PKA, PI3K/AKT, and M1/M2 microglia polarization. Eur J Pharmacol 952:175810. 10.1016/j.ejphar.2023.175810 37245858 10.1016/j.ejphar.2023.175810

[CR4] Bardoni R (2013) Role of presynaptic glutamate receptors in pain transmission at the spinal cord level. Curr Neuropharmacol 11:477–483. 10.2174/1570159X11311050002 24403871 10.2174/1570159X11311050002 PMC3763755

[CR5] Bennett R (2005) Fibromyalgia: present to future. Curr Rheumatol Rep 7:371–376. 10.1007/s11926-005-0022-y 16174485 10.1007/s11926-005-0022-y

[CR70] Boomershine S (2015) Fibromyalgia: the prototypical central sensitivity syndrome. Curr Rheumatol Rev 11:131–145. 10.3390/healthcare11020223 26088213 10.2174/1573397111666150619095007

[CR6] Brum EdaS, Fialho MFP, Fischer SPM et al (2020) Relevance of mitochondrial dysfunction in the reserpine-induced experimental fibromyalgia model. Mol Neurobiol 57:4202–4217. 10.1007/s12035-020-01996-1 32685997 10.1007/s12035-020-01996-1

[CR7] Brum ES, Becker G, Fialho MFP, Oliveira SM (2022) Animal models of fibromyalgia: what is the best choice? Pharmacol Ther 230:107959. 10.1016/j.pharmthera.2021.107959 34265360 10.1016/j.pharmthera.2021.107959

[CR101] Cabo-Meseguer A, Cerdá-Olmedo G, Trillo-Mata JL (2017) Fibromyalgia: prevalence, epidemiologic profiles and economic costs. Med Clin, 149(10):441–448. 10.1016/j.medcli.2017.06.008 10.1016/j.medcli.2017.06.008 28734619

[CR9] Cao B, Xu Q, Shi Y et al (2024) Pathology of pain and its implications for therapeutic interventions. Signal Transduct Target Ther 9:155. 10.1038/s41392-024-01845-w 38851750 10.1038/s41392-024-01845-w PMC11162504

[CR99] Carson JW, Carson KM, Jones KD, Bennett RM, Wright CL, Mist SD (2010) A pilot randomized controlled trial of the yoga of awareness program in the management of fibromyalgia. PAIN®, 151(2):530–539. 10.1016/j.pain.2010.08.020 10.1016/j.pain.2010.08.020 PMC5568071 20946990

[CR10] Castro LVG, Gonçalves-de-Albuquerque CF, Silva AR (2022) Polarization of microglia and its therapeutic potential in sepsis. Int J Mol Sci 23:4925. 10.3390/ijms23094925 35563317 10.3390/ijms23094925 PMC9101892

[CR8] Çakirgöz E, Durdaği G, Eser ÖZ (2025) Enhanced Analgesia: Synergistic Effects of Melatonin and Tramadol on Acute Thermal Nociception in Wistar Rats via Tail-Flick and Hot-Plate Tests. Behav Brain Res 115641. 10.1016/j.bbr.2025.115641 10.1016/j.bbr.2025.115641 40355030

[CR12] Chen G, Zhang Y-Q, Qadri YJ et al (2018) Microglia in pain: detrimental and protective roles in pathogenesis and resolution of pain. Neuron 100:1292–1311. 10.1016/j.neuron.2018.11.009 30571942 10.1016/j.neuron.2018.11.009 PMC6312407

[CR11] Cheng C-F, Cheng J-K, Chen C-Y, Rau R-H, Yu-Cheng Chang M-LT (2015) Nerve growth factor-induced synapse-like structures in contralateral sensory ganglia contribute to chronic mirror-image pain. Pain 156:2295–2309. 10.1097/j.pain.0000000000000280 26121254 10.1097/j.pain.0000000000000280

[CR13] Chiang RP, Huang C, Tsai Y (2013) Melatonin reduces median nerve injury-induced mechanical hypersensitivity via inhibition of microglial p38 mitogen‐activated protein kinase activation in rat cuneate nucleus. J Pineal Res 54:232–244. 10.1111/jpi.12029 23237358 10.1111/jpi.12029

[CR14] Culling CFA (2013) Handbook of histopathological and histochemical techniques: including museum techniques. Butterworth-Heinemann

[CR15] d’Mello R, Marchand F, Pezet S et al (2011) Perturbing PSD-95 interactions with NR2B-subtype receptors attenuates spinal nociceptive plasticity and neuropathic pain. Mol Ther 19:1780–1792. 10.1038/mt.2011.42 10.1038/mt.2011.42 PMC3188755 21427709

[CR16] Dagnino APA, da Silva RBM, Chagastelles PC et al (2019) Nociceptin/orphanin FQ receptor modulates painful and fatigue symptoms in a mouse model of fibromyalgia. Pain 160:1383–1401. 10.1097/j.pain.0000000000001513 30720581 10.1097/j.pain.0000000000001513

[CR17] Das A, Wallace IVG, Reiter RJ et al (2013) Overexpression of melatonin membrane receptors increases calcium-binding proteins and protects VSC4. 1 motoneurons from glutamate toxicity through multiple mechanisms. J Pineal Res 54:58–68. 10.1111/j.1600-079X.2012.01022.x 22823500 10.1111/j.1600-079X.2012.01022.x PMC11877314

[CR18] De la Luz-Cuellar YE, Rodríguez-Palma EJ, Franco-Enzástiga Ú et al (2019) Blockade of spinal α5-GABAA receptors differentially reduces reserpine-induced fibromyalgia-type pain in female rats. Eur J Pharmacol 858:172443. 10.1016/j.ejphar.2019.172443 10.1016/j.ejphar.2019.172443 31181208

[CR19] de Zanette SA, Vercelino R, Laste G et al (2014) Melatonin analgesia is associated with improvement of the descending endogenous pain-modulating system in fibromyalgia: a phase II, randomized, double-dummy, controlled trial. BMC Pharmacol Toxicol 15:1–14. 10.1186/2050-6511-15-40 25052847 10.1186/2050-6511-15-40 PMC4119581

[CR20] Deng M, Chen S-R, Pan H-L (2019) Presynaptic NMDA receptors control nociceptive transmission at the spinal cord level in neuropathic pain. Cell Mol Life Sci 76:1889–1899. 10.1007/s00018-019-03047-y 30788514 10.1007/s00018-019-03047-y PMC6482077

[CR21] Deuis JR, Dvorakova LS, Vetter I (2017) Methods used to evaluate pain behaviors in rodents. Front Mol Neurosci 10:284. 10.3389/fnmol.2017.00284 28932184 10.3389/fnmol.2017.00284 PMC5592204

[CR22] Domercq M, Vázquez-Villoldo N, Matute C (2013) Neurotransmitter signaling in the pathophysiology of microglia. Front Cell Neurosci 7:49. 10.3389/fncel.2013.00049 23626522 10.3389/fncel.2013.00049 PMC3630369

[CR23] El-Sawaf ES, El Maraghy NN, El-Abhar HS et al (2024) Melatonin mitigates vincristine-induced peripheral neuropathy by inhibiting TNF-α/astrocytes/microglial cells activation in the spinal cord of rats, while preserving vincristine’s chemotherapeutic efficacy in lymphoma cells. Toxicol Appl Pharmacol 492:117134. 10.1016/j.taap.2024.117134 39461624 10.1016/j.taap.2024.117134

[CR24] Espinoza N, Papadopoulos V (2025) Role of mitochondrial dysfunction in neuropathy. Int J Mol Sci. 10.3390/ijms26073195 40243998 10.3390/ijms26073195 PMC11989173

[CR26] Favero G, Trapletti V, Bonomini F et al (2017) Oral supplementation of melatonin protects against fibromyalgia-related skeletal muscle alterations in reserpine-induced myalgia rats. Int J Mol Sci 18:1389. 10.3390/ijms18071389 28661421 10.3390/ijms18071389 PMC5535882

[CR25] Favero G, Bonomini F, Franco C, Rezzani R (2019) Mitochondrial dysfunction in skeletal muscle of a fibromyalgia model: the potential benefits of melatonin. Int J Mol Sci 20:765. 10.3390/ijms20030765 30754674 10.3390/ijms20030765 PMC6386947

[CR27] Ferrari LF, Lotufo CM, Araldi D et al (2014) Inflammatory sensitization of nociceptors depends on activation of NMDA receptors in DRG satellite cells. Proc Natl Acad Sci U S A 111:18363–18368. 10.1073/pnas.1420601111 25489099 10.1073/pnas.1420601111 PMC4280647

[CR28] Fitzcharles M-A, Cohen SP, Clauw DJ et al (2021) Nociplastic pain: towards an understanding of prevalent pain conditions. Lancet 397:2098–2110. 10.1016/S0140-6736(21)00392-5 34062144 10.1016/S0140-6736(21)00392-5

[CR29] Flatters SJL (2015) The contribution of mitochondria to sensory processing and pain. Prog Mol Biol Transl Sci 131:119–146. 10.1016/bs.pmbts.2014.12.004 25744672 10.1016/bs.pmbts.2014.12.004

[CR30] Fusco R, Siracusa R, D’Amico R et al (2019) Melatonin plus folic acid treatment ameliorates reserpine-induced fibromyalgia: an evaluation of pain, oxidative stress, and inflammation. Antioxidants 8:628. 10.3390/antiox8120628 31817734 10.3390/antiox8120628 PMC6943570

[CR31] Galley HF, McCormick B, Wilson KL et al (2017) Melatonin limits paclitaxel-induced mitochondrial dysfunction in vitro and protects against paclitaxel‐induced neuropathic pain in the rat. J Pineal Res 63:e12444. 10.1111/jpi.12444 28833461 10.1111/jpi.12444 PMC5656911

[CR32] Gao J, Su G, Liu J et al (2020) Mechanisms of inhibition of excessive microglial activation by melatonin. J Mol Neurosci 70:1229–1236. 10.1007/s12031-020-01531-w 32222896 10.1007/s12031-020-01531-w

[CR33] Gröne E, Crispin A, Fleckenstein J et al (2012) Test order of quantitative sensory testing facilitates mechanical hyperalgesia in healthy volunteers. J Pain 13:73–80. 10.1016/j.jpain.2011.10.005 22208803 10.1016/j.jpain.2011.10.005

[CR34] Gu N, Yi M-H, Murugan M et al (2022) Spinal microglia contribute to sustained inflammatory pain via amplifying neuronal activity. Mol Brain 15:86. 10.1186/s13041-022-00970-3 36289499 10.1186/s13041-022-00970-3 PMC9609165

[CR35] Gui W-S, Wei X, Mai C-L et al (2016) Interleukin-1β overproduction is a common cause for neuropathic pain, memory deficit, and depression following peripheral nerve injury in rodents. Mol Pain. 10.1177/1744806916646784 27175012 10.1177/1744806916646784 PMC4956151

[CR36] Gyorfi M, Rupp A, Abd-Elsayed A (2022) Fibromyalgia pathophysiology. Biomedicines 10:3070. 10.3390/biomedicines10123070 36551826 10.3390/biomedicines10123070 PMC9776089

[CR37] Ibrahim SM, Kamel AS, Ahmed KA et al (2024) The preferential effect of clemastine on F3/Contactin-1/Notch-1 compared to Jagged-1/Notch-1 justifies its remyelinating effect in an experimental model of multiple sclerosis in rats. Int Immunopharmacol 128:111481. 10.1016/j.intimp.2023.111481 38232534 10.1016/j.intimp.2023.111481

[CR38] Ikeda N, Kawasaki M, Baba K et al (2023) Chemogenetic activation of oxytocin neurons improves pain in a Reserpine-induced fibromyalgia rat model. Neuroscience 528:37–53. 10.1016/j.neuroscience.2023.07.028 37532013 10.1016/j.neuroscience.2023.07.028

[CR39] Ito D, Imai Y, Ohsawa K et al (1998) Microglia-specific localisation of a novel calcium binding protein, Iba1. Mol Brain Res 57:1–9. 10.1016/s0169-328x(98)00040-0 9630473 10.1016/s0169-328x(98)00040-0

[CR40] Jang K, Garraway SM (2024) A review of dorsal root ganglia and primary sensory neuron plasticity mediating inflammatory and chronic neuropathic pain. Neurobiol Pain 15:100151. 10.1016/j.ynpai.2024.100151 38314104 10.1016/j.ynpai.2024.100151 PMC10837099

[CR41] Jenwitheesuk A, Boontem P, Wongchitrat P et al (2017) Melatonin regulates the aging mouse hippocampal homeostasis via the sirtuin1-FOXO1 pathway. EXCLI J 16:340–353. 10.17179/excli2016-852 28507478 10.17179/excli2016-852 PMC5427465

[CR42] Ji R-R, Nackley A, Huh Y et al (2018) Neuroinflammation and central sensitization in chronic and widespread pain. Anesthesiology 129:343. 10.1097/ALN.0000000000002130 29462012 10.1097/ALN.0000000000002130 PMC6051899

[CR43] Jia N, Sun Q, Su Q, Chen G (2016) SIRT1-mediated deacetylation of PGC1α attributes to the protection of curcumin against glutamate excitotoxicity in cortical neurons. Biochem Biophys Res Commun 478:1376–1381. 10.1016/j.bbrc.2016.08.132 27568287 10.1016/j.bbrc.2016.08.132

[CR44] Jung C, Ichesco E, Ratai E-M et al (2020) Magnetic resonance imaging of neuroinflammation in chronic pain: a role for astrogliosis? Pain 161:1555–1564. 10.1097/j.pain.0000000000001815 31990749 10.1097/j.pain.0000000000001815 PMC7305954

[CR45] Jung Y-H, Kim H, Lee D et al (2021) Dysfunctional energy metabolisms in fibromyalgia compared with healthy subjects. Mol Pain 17:17448069211012832. 10.1177/17448069211012833 10.1177/17448069211012833 PMC8113919 33940974

[CR46] Kamaly NA, Kamel AS, Sadik NA, Shahin NN (2025) Milnacipran and vanillin alleviate fibromyalgia-associated depression in reserpine-induced rat model: role of Wnt/β-catenin signaling. Mol Neurobiol. 10.1007/s12035-025-04723-w 39924579 10.1007/s12035-025-04723-w PMC12078381

[CR47] Kawasaki Y, Zhang L, Cheng J-K, Ji R-R (2008) Cytokine mechanisms of central sensitization: distinct and overlapping role of interleukin-1beta, interleukin-6, and tumor necrosis factor-alpha in regulating synaptic and neuronal activity in the superficial spinal cord. J Neurosci 28:5189–5194 18480275 10.1523/JNEUROSCI.3338-07.2008 PMC2408767

[CR48] Kohno K, Tsuda M (2025) Neuron–microglia interactions modulating neuropathic pain. Int Immunol dxaf022. 10.1093/intimm/dxaf022 10.1093/intimm/dxaf022 40251994

[CR49] Kung L-H, Gong K, Adedoyin M et al (2013) Evidence for glutamate as a neuroglial transmitter within sensory ganglia. PLoS One 8:e68312 23844184 10.1371/journal.pone.0068312 PMC3699553

[CR50] Latremoliere A, Woolf CJ (2009) Central sensitization: a generator of pain hypersensitivity by central neural plasticity. J Pain 10:895–926 19712899 10.1016/j.jpain.2009.06.012 PMC2750819

[CR51] Laurido C, Pelissier T, Soto-Moyano R et al (2002) Effect of melatonin on rat spinal cord nociceptive transmission. Neuroreport 13:89–91 11924900 10.1097/00001756-200201210-00021

[CR52] Li A, Huang C-J, Gu K-P et al (2022) PSD-95 in the anterior cingulate cortex contributes to neuropathic pain by interdependent activation with NR2B. Sci Rep 12:17114 36224339 10.1038/s41598-022-21488-7 PMC9556829

[CR53] Ling-Jun Xu MD, Jing Wang MD, Yu-Dan Li BS, Xing-Hui He BS (2023) Reduction of SIRT1-mediated epigenetic upregulation of Nav1. 7 contributes to oxaliplatin-induced neuropathic pain. Pain Physician 26:E213 37192244

[CR54] Liu XJ, Salter MW (2010) Glutamate receptor phosphorylation and trafficking in pain plasticity in spinal cord dorsal horn. Eur J Neurosci 32:278–289 20629726 10.1111/j.1460-9568.2010.07351.x PMC3589563

[CR55] Lv C, Hu H-Y, Zhao L et al (2015) Intrathecal SRT1720, a SIRT1 agonist, exerts anti-hyperalgesic and anti-inflammatory effects on chronic constriction injury-induced neuropathic pain in rats. Int J Clin Exp Med 8:7152 26221253 PMC4509198

[CR56] Macchi C, Giachi A, Fichtner I et al (2024) Mitochondrial function in patients affected with fibromyalgia syndrome is impaired and correlates with disease severity. Sci Rep 14:1–9. 10.1038/s41598-024-81298-x 39632893 10.1038/s41598-024-81298-x PMC11618515

[CR57] Martínez-Lavín M (2022) Centralized nociplastic pain causing fibromyalgia: an emperor with no cloths? Clin Rheumatol 41:3915–3917. 10.1007/s10067-022-06407-5 36239845 10.1007/s10067-022-06407-5 PMC9561334

[CR58] Meeus M, Nijs J, Hermans L et al (2013) The role of mitochondrial dysfunctions due to oxidative and nitrosative stress in the chronic pain or chronic fatigue syndromes and fibromyalgia patients: peripheral and central mechanisms as therapeutic targets? Expert Opin Ther Targets 17:1081–1089. 10.1517/14728222.2013.818657 23834645 10.1517/14728222.2013.818657

[CR59] Merlo S, Luaces JP, Spampinato SF et al (2020) *SIRT1* mediates melatonin’s effects on microglial activation in hypoxia: in vitro and in vivo evidence. Biomolecules 10:364. 10.3390/biom10030364 32120833 10.3390/biom10030364 PMC7175216

[CR60] Mohamed MM, Zaki HF, Kamel AS (2025) Possible interaction of suramin with thalamic P2X receptors and *NLRP3* inflammasome activation alleviates reserpine-induced fibromyalgia-like symptoms. J Neuroimmune Pharmacol 20:1–22. 10.1007/s11481-025-10207-4 10.1007/s11481-025-10207-4 PMC12055955 40329125

[CR62] Nagakura Y, Oe T, Aoki T, Matsuoka N (2009) Biogenic amine depletion causes chronic muscular pain and tactile allodynia accompanied by depression: a putative animal model of fibromyalgia. Pain 146:26–33. 10.1016/j.pain.2009.05.024 19646816 10.1016/j.pain.2009.05.024

[CR61] Nagakura Y, Miwa M, Yoshida M et al (2019) Spontaneous pain-associated facial expression and efficacy of clinically used drugs in the reserpine-induced rat model of fibromyalgia. Eur J Pharmacol 864:172716. 10.1016/j.ejphar.2019.172716 31589868 10.1016/j.ejphar.2019.172716

[CR63] Niciu MJ, Kelmendi B, Sanacora G (2012) Overview of glutamatergic neurotransmission in the nervous system. Pharmacol Biochem Behav 100:656–664. 10.1016/j.pbb.2011.08.008 21889952 10.1016/j.pbb.2011.08.008 PMC3253893

[CR64] Noseda R, Hernández A, Valladares L et al (2004) Melatonin-induced inhibition of spinal cord synaptic potentiation in rats is *MT2* receptor-dependent. Neurosci Lett 360:41–44. 10.1016/j.neulet.2004.01.080 15082174 10.1016/j.neulet.2004.01.080

[CR65] Pereira V, Goudet C (2019) Emerging trends in pain modulation by metabotropic glutamate receptors. Front Mol Neurosci 11:464. 10.3389/fnmol.2018.00464 30662395 10.3389/fnmol.2018.00464 PMC6328474

[CR66] Qi J, Chen C, Meng Q-X et al (2016) Crosstalk between activated microglia and neurons in the spinal dorsal horn contributes to stress-induced hyperalgesia. Sci Rep 6:39442. 10.1038/srep39442 27995982 10.1038/srep39442 PMC5171842

[CR67] Qin S, Yang C, Huang W et al (2018) Sulforaphane attenuates microglia-mediated neuronal necroptosis through down-regulation of MAPK/NF-κB signaling pathways in LPS-activated BV-2 microglia. Pharmacol Res 133:218–235. 10.1016/j.phrs.2018.01.014 29391237 10.1016/j.phrs.2018.01.014

[CR68] Rasheed MZ, Andrabi SS, Salman M et al (2018) Melatonin improves behavioral and biochemical outcomes in a rotenone-induced rat model of Parkinson’s disease. J Environ Pathol Toxicol Oncol. 10.1615/JEnvironPatholToxicolOncol.2018025666 30055549 10.1615/JEnvironPatholToxicolOncol.2018025666

[CR69] Romano IR, D’Angeli F, Gili E et al (2024) Melatonin enhances neural differentiation of adipose-derived mesenchymal stem cells. Int J Mol Sci. 10.3390/ijms25094891 38732109 10.3390/ijms25094891 PMC11084714

[CR100] Ruschak I, Montesó-Curto P, Rosselló L, Martín CA, Sánchez-Montesó L, Toussaint L, Ruschak I, Montesó-Curto P, Rosselló L, Martín CA, Sánchez-Montesó L, Toussaint L (2023) Fibromyalgia syndrome pain in men and women: a scoping review. Healthcare, 11(2). 10.3390/healthcare11020223 10.3390/healthcare11020223 PMC9859454 36673591

[CR71] Sarzi-Puttini P, Giorgi V, Marotto D, Atzeni F (2020) Fibromyalgia: an update on clinical characteristics, aetiopathogenesis and treatment. Nat Rev Rheumatol 16:645–660. 10.1038/s41584-020-00506-w 33024295 10.1038/s41584-020-00506-w

[CR72] Shafiek MZ, Zaki HF, Mohamed AF (2025a) New ways to repurpose salmeterol in an animal model of fibromyalgia. Fundam Clin Pharmacol 39:e13041. 10.1111/fcp.13041 39496328 10.1111/fcp.13041

[CR73] Shafiek MZ, Zaki HF, Mohamed AF, Ibrahim WW (2025b) Novel trajectories towards possible effects of semaglutide for amelioration of reserpine-induced fibromyalgia in rats: contribution of cAMP/PKA/p-CREB and M1/M2 microglia polarization. J Neuroimmune Pharmacol 20:43. 10.1007/s11481-025-10196-4 40240584 10.1007/s11481-025-10196-4 PMC12003577

[CR74] Siracusa R, Paola R Di, Cuzzocrea S, Impellizzeri D (2021) Fibromyalgia: pathogenesis, mechanisms, diagnosis and treatment options update. Int J Mol Sci 22:3891. 10.3390/ijms22083891 33918736 10.3390/ijms22083891 PMC8068842

[CR75] Srinivasan V, Lauterbach EC, Yu Ho K et al (2012) Melatonin in antinociception: its therapeutic applications. Curr Neuropharmacol 10:167–178. 10.2174/157015912800604489 23204986 10.2174/157015912800604489 PMC3386506

[CR76] Staud R, Domingo M (2001) Evidence for abnormal pain processing in fibromyalgia syndrome. Pain Med 2:208–215. 10.1007/s11926-011-0206-6 15102253 10.1046/j.1526-4637.2001.01030.x

[CR77] Sun T, Xiao H-S, Zhou P-B et al (2006) Differential expression of synaptoporin and synaptophysin in primary sensory neurons and up-regulation of synaptoporin after peripheral nerve injury. Neuroscience 141:1233–1245. 10.1016/j.neuroscience.2006.05.010 16777346 10.1016/j.neuroscience.2006.05.010

[CR78] Tancheva L, Lazarova M, Saso L et al (2021) Beneficial effect of melatonin on motor and memory disturbances in 6-OHDA-lesioned rats. J Mol Neurosci 71:702–712. 10.1007/s12031-020-01760-z 33403591 10.1007/s12031-020-01760-z

[CR79] Tanei S, Miwa M, Yoshida M et al (2020) The method simulating spontaneous pain in patients with nociplastic pain using rats with fibromyalgia-like condition. MethodsX 7:100826. 10.1016/j.mex.2020.100826 32195142 10.1016/j.mex.2020.100826 PMC7078388

[CR80] Temmermand R, Barrett JE, Fontana ACK (2022) Glutamatergic systems in neuropathic pain and emerging non-opioid therapies. Pharmacol Res 185:106492. 10.1016/j.phrs.2022.106492 36228868 10.1016/j.phrs.2022.106492 PMC10413816

[CR81] Tu Y, Sun R-Q, Willis WD (2004) Effects of intrathecal injections of melatonin analogs on capsaicin-induced secondary mechanical allodynia and hyperalgesia in rats. Pain 109:340–350. 10.1016/j.pain.2004.01.027 15157695 10.1016/j.pain.2004.01.027

[CR82] Turan Yücel N, Üçel Uİ, Demir Özkay Ü et al (2023) Effect of reboxetine treatment on BDNF, synaptophysin, and PSD-95 levels in the spinal dorsal horn of rats with diabetic neuropathy. Clin Exp Health Sci 13:710–718. 10.33808/clinexphealthsci.1222028

[CR83] Vanderwall AG, Milligan ED (2019) Cytokines in pain: harnessing endogenous anti-inflammatory signaling for improved pain management. Front Immunol 10:3009. 10.3389/fimmu.2019.03009 31921220 10.3389/fimmu.2019.03009 PMC6935995

[CR84] Wang X, Sirianni A, Pei Z et al (2011) The melatonin MT1 receptor axis modulates mutant Huntingtin-mediated toxicity. J Neurosci 31:14496–14507. 10.1523/JNEUROSCI.3059-11.2011 21994366 10.1523/JNEUROSCI.3059-11.2011 PMC3213696

[CR98] Wolfe F, Clauw DJ, Fitzcharles MA, Goldenberg DL, Katz RS, Mease P, Russell AS, Russell IJ, Winfield JB, Yunus MB (2010) The american college of rheumatology preliminary diagnostic criteria for fibromyalgia and measurement of symptom severity. Arthritis Care Res, 62(5):600–610. 10.1002/acr.20140 10.1002/acr.20140 20461783

[CR85] Xiong Z, Peng G, Deng J et al (2024) Therapeutic targets and potential delivery systems of melatonin in osteoarthritis. Front Immunol 15:1331934. 10.3389/fimmu.2024.1331934 38327517 10.3389/fimmu.2024.1331934 PMC10847247

[CR86] Yam MF, Loh YC, Tan CS et al (2018) General pathways of pain sensation and the major neurotransmitters involved in pain regulation. Int J Mol Sci 19:2164. 10.3390/ijms19082164 30042373 10.3390/ijms19082164 PMC6121522

[CR87] Yang X, Xu S, Qian Y, Xiao Q (2017) Resveratrol regulates microglia M1/M2 polarization via PGC-1α in conditions of neuroinflammatory injury. Brain Behav Immun 64:162–172. 10.1016/j.bbi.2017.03.003 28268115 10.1016/j.bbi.2017.03.003

[CR88] Yao X, Li L, Kandhare AD et al (2020) Attenuation of reserpine-induced fibromyalgia via ROS and serotonergic pathway modulation by fisetin, a plant flavonoid polyphenol. Exp Ther Med 19:1343–1355. 10.3892/etm.2019.8328 32010308 10.3892/etm.2019.8328 PMC6966137

[CR89] Yilmaz U, Tanbek K, Gul S et al (2023) Melatonin attenuates cerebral ischemia/reperfusion injury through inducing autophagy. Neuroendocrinology 113:1035–1050. 10.1159/000531567 37321200 10.1159/000531567

[CR90] Yu H, Nagi SS, Usoskin D et al (2024) Leveraging deep single-soma RNA sequencing to explore the neural basis of human somatosensation. Nat Neurosci 27:2326–2340. 10.1038/s41593-024-01794-1 39496796 10.1038/s41593-024-01794-1 PMC11614738

[CR91] Zatroch KK, Knight CG, Reimer JN, Pang DSJ (2016) Refinement of intraperitoneal injection of sodium pentobarbital for euthanasia in laboratory rats (*Rattus norvegicus*). BMC Vet Res 13:1–7. 10.1186/s12917-017-0982-y 10.1186/s12917-017-0982-y PMC5320784 28222732

[CR92] Zeng Y, Fang Q, Chen J et al (2023) Melatonin improves mitochondrial dysfunction and attenuates neuropathic pain by regulating SIRT1 in dorsal root ganglions. Neuroscience 534:29–40. 10.1016/j.neuroscience.2023.10.005 37832908 10.1016/j.neuroscience.2023.10.005

[CR95] Zhang Y, Cook A, Kim J et al (2013) Melatonin inhibits the caspase-1/cytochrome c/caspase-3 cell death pathway, inhibits MT1 receptor loss and delays disease progression in a mouse model of amyotrophic lateral sclerosis. Neurobiol Dis 55:26–35. 10.1016/j.nbd.2013.03.008 23537713 10.1016/j.nbd.2013.03.008 PMC3652329

[CR94] Zhang T, Xue R, Zhu L et al (2016) Evaluation of the analgesic effects of ammoxetine, a novel potent serotonin and norepinephrine reuptake inhibitor. Acta Pharmacol Sin 37:1154–1165. 10.1038/aps.2016.45 27424654 10.1038/aps.2016.45 PMC5022096

[CR93] Zhang J-D, Zhong Z-A, Xing W-Y (2025) Environmental enrichment for neuropathic pain via modulation of neuroinflammation. Front Mol Neurosci 18:1547647. 10.3389/fnmol.2025.1547647 40190342 10.3389/fnmol.2025.1547647 PMC11968435

[CR96] Zhao H, Zhang T, Zhang H et al (2024) Exercise-with-melatonin therapy improves sleep disorder and motor dysfunction in a rat model of ischemic stroke. Neural Regen Res 19:1336–1343. 10.4103/1673-5374.385844 37905883 10.4103/1673-5374.385844 PMC11467917

[CR97] Zhou H-Y, Chen S-R, Pan H-L (2011) Targeting n-methyl-D-aspartate receptors for treatment of neuropathic pain. Expert Rev Clin Pharmacol 4:379–388. 10.1586/ecp.11.17 21686074 10.1586/ecp.11.17 PMC3113704

