# Supplementary figures and images for: Melatonin Mitigates Central Sensitization and Nociplastic Pain in Spinal Cord and Dorsal Root Ganglia of FM Rat Model: Modulation of SIRT1/PGC-1α/MAPK/NF-κB Signaling
Source: J Neuroimmune Pharmacol. 2026 Feb 13;21(1):12. doi: 10.1007/s11481-025-10274-7 (PMC12904926; doi:10.1007/s11481-025-10274-7)

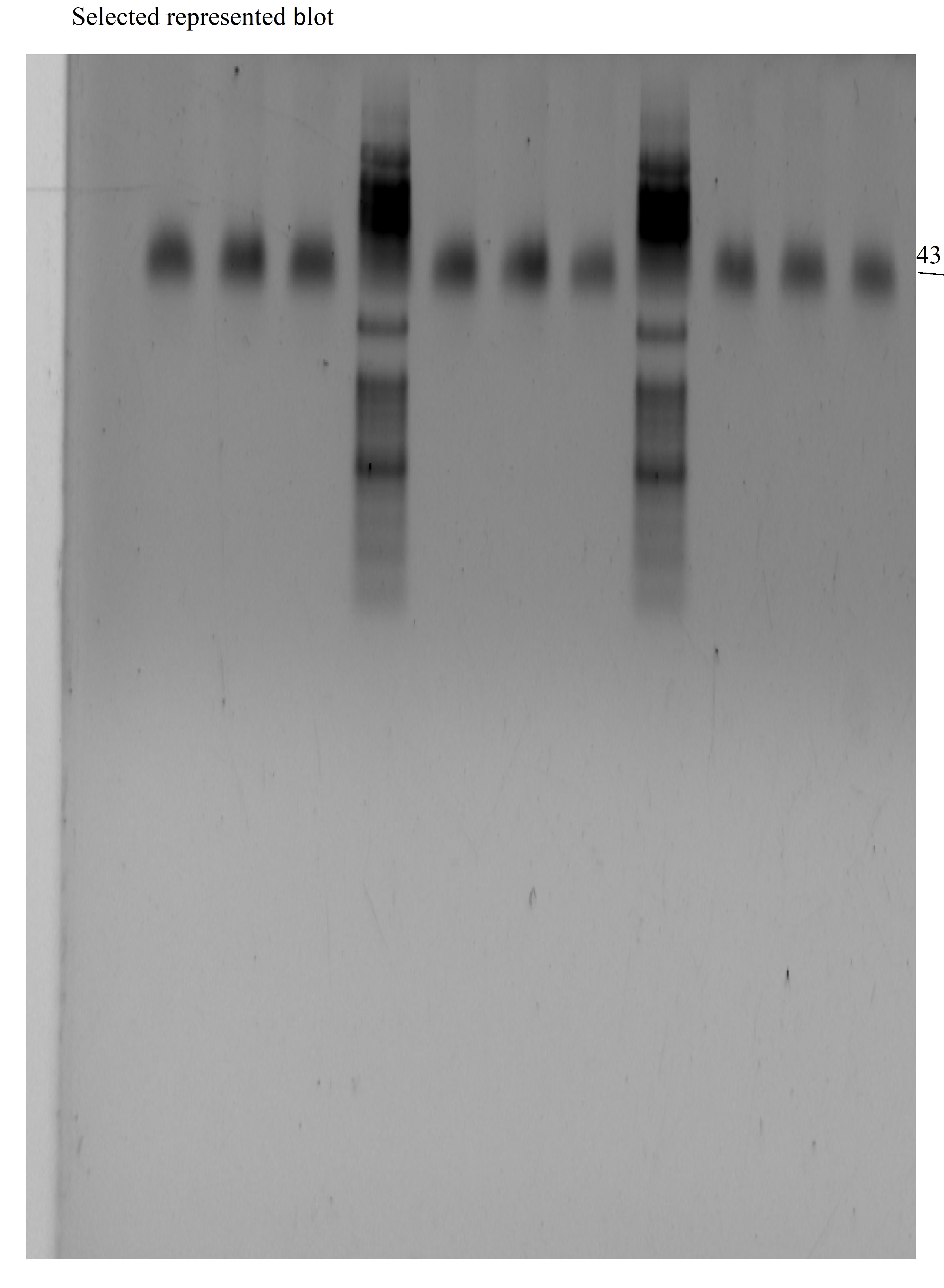

Supplement: Supplementary file 1 — Supplementary Material 1 (JPG.926 KB) [file 11481_2025_10274_MOESM1_ESM.jpg]

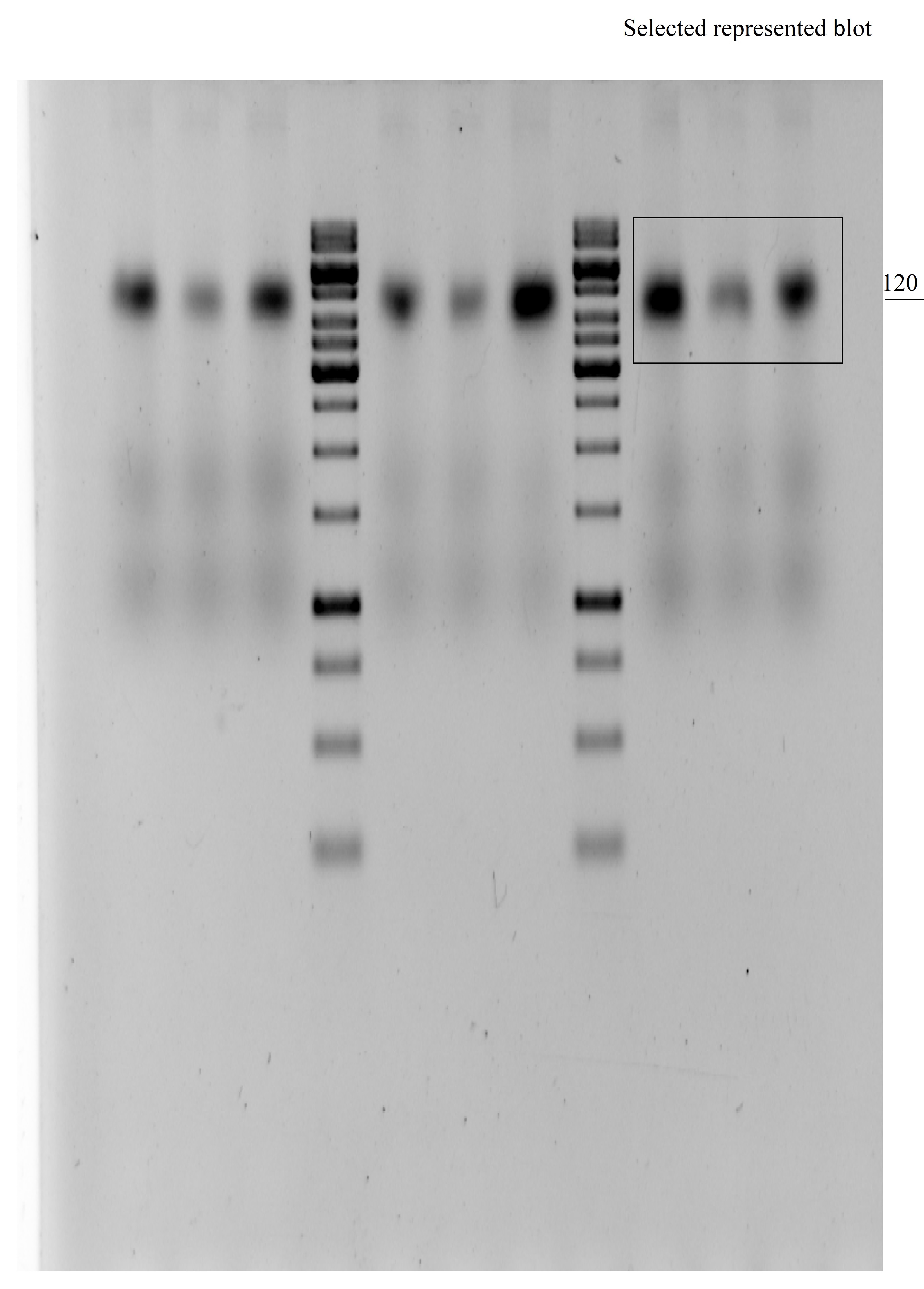

Supplement: Supplementary file 2 — Supplementary Material 2 (JPG.1.08 MB) [file 11481_2025_10274_MOESM2_ESM.jpg]

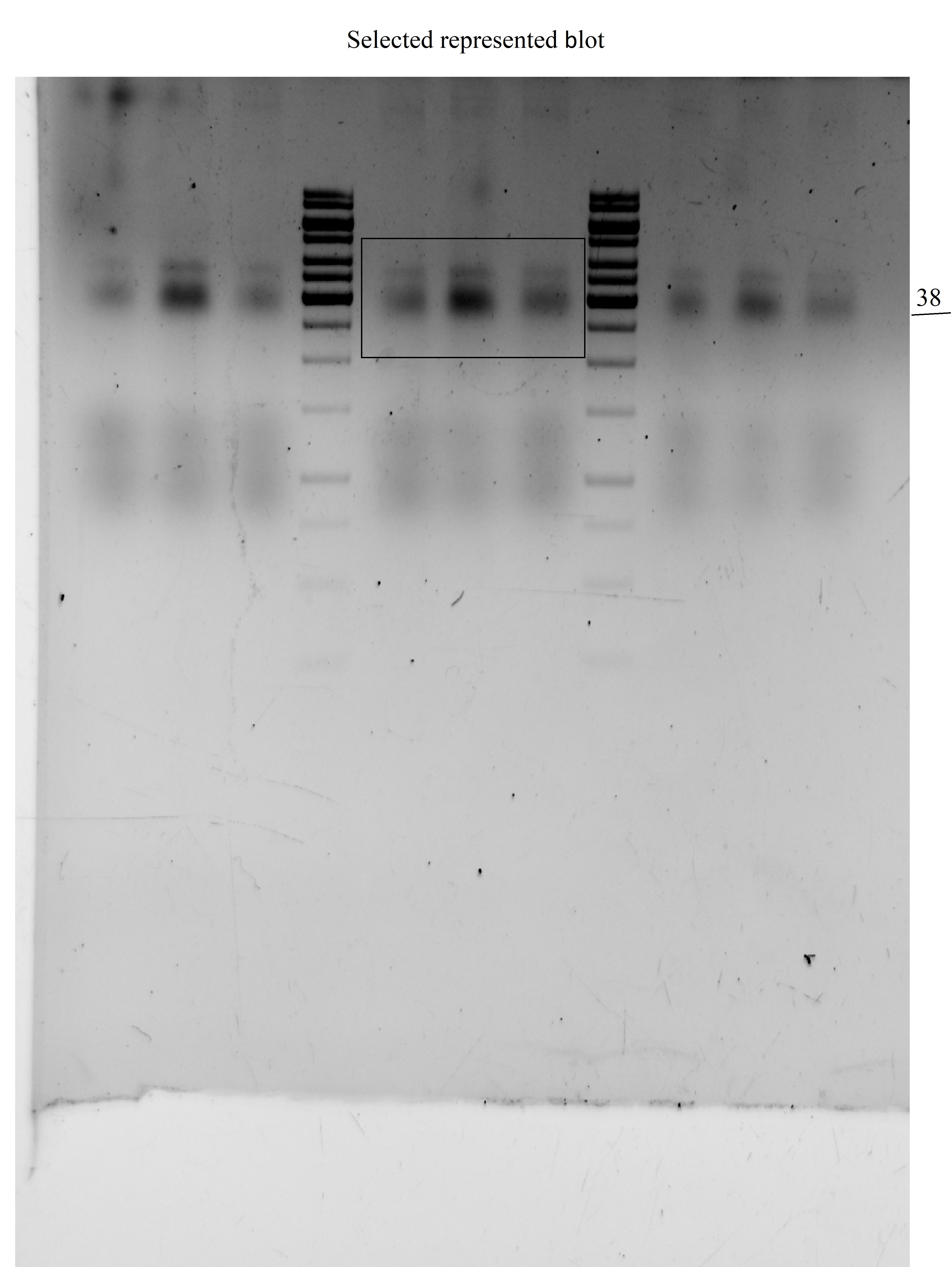

Supplement: Supplementary file 3 — Supplementary Material 3 (JPG.1.17 MB) [file 11481_2025_10274_MOESM3_ESM.jpg]

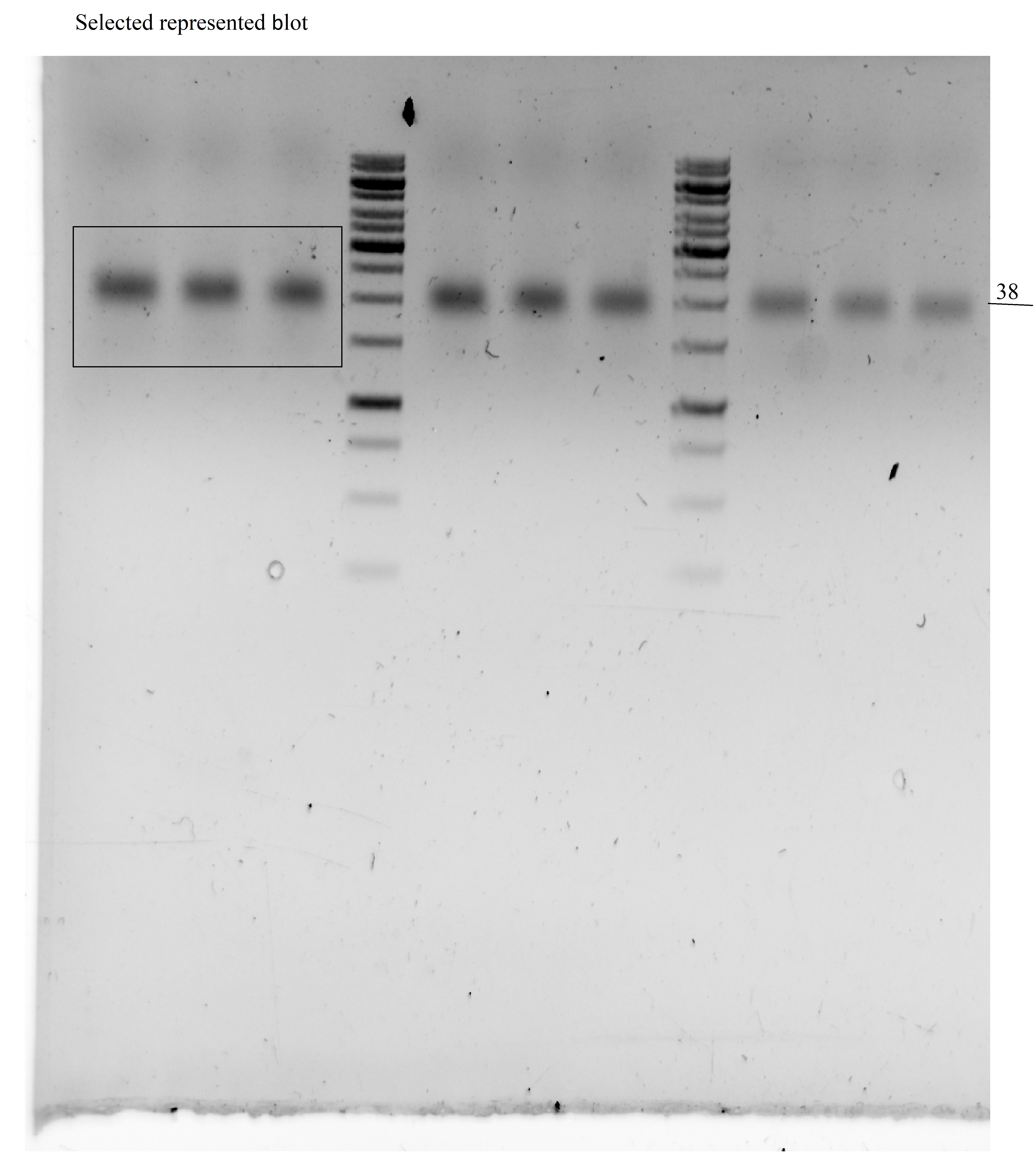

Supplement: Supplementary file 4 — Supplementary Material 4 (JPG.1.27 MB) [file 11481_2025_10274_MOESM4_ESM.jpg]
